# Beyond DNA sensing: expanding the role of cGAS/STING in immunity and diseases

**DOI:** 10.1007/s12272-023-01452-3

**Published:** 2023-06-24

**Authors:** Jin Kyung Seok, Minhyuk Kim, Han Chang Kang, Yong-Yeon Cho, Hye Suk Lee, Joo Young Lee

**Affiliations:** College of Pharmacy, The Catholic University of Korea, Bucheon, 14662 Republic of Korea

**Keywords:** cGAS, STING, DNA, immunity, Inflammation, Cancer

## Abstract

Cyclic guanosine monophosphate-adenosine monophosphate (cGAMP) synthase (cGAS) is a DNA sensor that elicits a robust type I interferon response by recognizing ubiquitous danger-associated molecules. The cGAS/stimulator of interferon genes (cGAS/STING) is activated by endogenous DNA, including DNA released from mitochondria and extranuclear chromatin, as well as exogenous DNA derived from pathogenic microorganisms. cGAS/STING is positioned as a key axis of autoimmunity, the inflammatory response, and cancer progression, suggesting that the cGAS/STING signaling pathway represents an efficient therapeutic target. Based on the accumulated evidence, we present insights into the prevention and treatment of cGAS/STING-related chronic immune and inflammatory diseases. This review presents the current state of clinical and nonclinical development of modulators targeting cGAS/STING, providing useful information on the design of therapeutic strategies.

## Introduction

The activation of innate and adaptive immunity is initiated by the detection of microbial infection by pattern-recognition receptors (PRRs) (Janeway and Medzhitov 2002). The families of PRRs include Toll-like receptors (TLRs) (Takeda and Akira 2005), retinoic acid-inducible gene I-like receptors (RLRs) (Rehwinkel and Gack 2020), NOD-like receptors (NLRs) (Inohara and Nunez 2003), and C-type lectin-like receptors (CLRs) (Ebner et al. 2003). PRRs recognize pathogen-associated molecular patterns (PAMPs) derived from invading pathogenic microbes as well as danger-associated molecular patterns (DAMPs) released from damaged tissues and cells (Seok et al. 2021). PAMPs and DAMPs include various molecules composed of polysaccharides, lipids, fatty acids, peptides, and nucleic acids (Jounai et al. 2013).

DNA derived from pathogenic microbes or host cells is detected by various PRRs, such as TLR9, DNA-dependent activator of IRFs (DAI), LRR binding FLII interacting protein 1 (LRRFIP1), DExD/H box helicases (DDX41), absent in melanoma 2 (AIM2), and interferon-inducible protein 16 (IFI16), culminating in production of the type I interferons, IL-1β and IL-18. The role of these PRRs in inducing DNA-dependent immunity is somewhat limited because they are activated in a DNA sequence-specific or cell type-dependent manner. Cyclic GMP–AMP (cGAMP) synthase (cGAS) is considered a more universal cytosolic DNA sensor because cGAS responds to DNA in a DNA-sequence-independent manner in a variety of cell types (Sun et al. 2013). cGAS senses cytosolic DNA derived from not only viruses and bacteria but also host DNA, such as mitochondrial DNA (mtDNA) and nuclear DNA, suggesting that it plays a critical role in regulating immunity and the host damage repair system. Accumulating evidence indicates that cGAS is important in inducing and controlling immunity, thereby affecting the progress and severity of immune disorders and inflammation-mediated diseases (Li and Chen 2018). In this review, we discuss therapeutic approaches to manipulate the activity of the cGAS/STING pathway for the prevention and treatment of chronic immune-related and inflammatory diseases.

## cGAS activation and signaling pathways

Human cGAS (also known as C6orf150, or male abnormal 21 domain containing 1 (MAB21D1)) is composed of 522 amino acids. cGAS recognizes pathogenic DNA from DNA and RNA viruses, activating innate immune cells and inducing essential immune responses against infection (Sun et al. 2013). cGAS is activated by host DNA, including nuclear, mitochondrial, and oxidized DNA, which reflect cellular damage and stress, thus highlighting its significant role in self-initiated immune-related diseases as well as host defense and tissue repair (Gao et al. 2015).

**Fig. 1:**
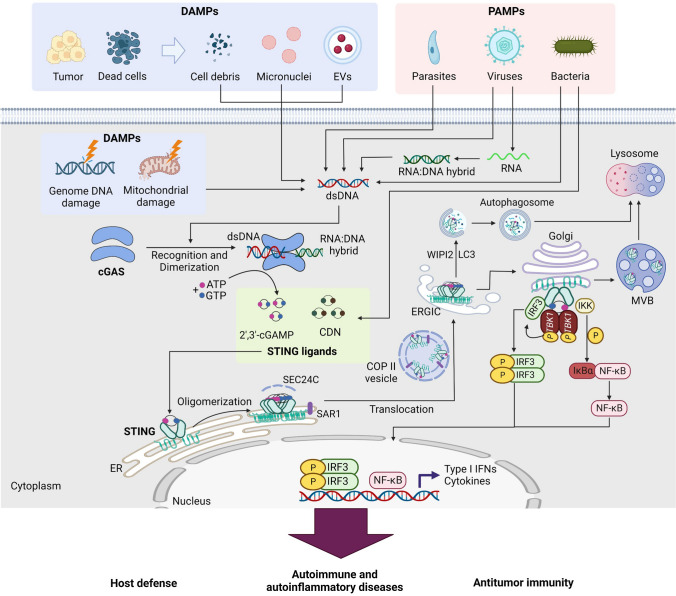
Activation of the cGAS/STING pathway by microbial DNA and self-DNA. Double-stranded DNA (dsDNA) derived from various sources of damage-associated molecular patterns (DAMPs), such as damaged cells and cancer cells, as well as dsDNA from various pathogen-associated molecular patterns (PAMPs) and RNA:DNA hybrids, activate the enzyme cyclic GMP-AMP synthase (cGAS) to synthesize 2′,3′-cyclic GMP-AMP (cGAMP), which serves as a STING ligand. Additionally, bacterial-derived cyclic dinucleotides (CDNs) act as STING ligands. Upon binding of STING ligands, STING translocates from the endoplasmic reticulum (ER) to the ER-Golgi intermediate compartment (ERGIC) via a process triggered by STING oligomerization and dependent on the SAR1 and SEC24C components. Within ERGIC, cGAMP-bound STING serves as a membrane source for the recruitment and lipidation of LC3 through a mechanism that is dependent on WIPI2. The resulting LC3-positive membranes then target DNA and pathogens to autophagosomes, which subsequently fuse with lysosomes. During translocation from the ERGIC to the Golgi, STING recruits TANK-binding kinase 1 (TBK1) and IκB kinase (IKK). This leads to phosphorylation of IRF3, which dimerizes and translocates to the nucleus to activate transcription of genes encoding type I interferons, including interferon-β (IFN-β). Phosphorylation of IκBα translocates NF-κB to the nucleus, where it activates the transcription of genes encoding proinflammatory cytokines, such as IL-6 and tumor necrosis factor (TNF). Finally, cGAMP-bound STING can also translocate to lysosomes for degradation via the multivesicular body (MVB) pathway, which involves the Golgi and endosomes

Recognition of DNA by cGAS is dependent on DNA length. Double-stranded DNA (dsDNA) longer than 20 bp activates cGAS, inducing dimerization of cGAS and resulting in the formation of a 2:2 DNA/cGAS complex, whereas dsDNA less than 20 bp is not able to induce cGAS dimerization and activation (Andreeva et al. 2017). cGAS comprises an unstructured N-terminal domain (amino acids 1–160) and a highly conserved C-terminal domain (amino acids 161–522). The N-terminal domain is not well conserved, with many K/R residues participating in the attachment of cGAS to the plasma membrane and its binding to DNA. The C-terminal region has two highly conserved motifs: a nucleotidyltransferase (NTase) core domain (160–330) and a Mab21 domain with zinc-ribbon insertion (213–513). The NTase domain is crucial for cGAS enzyme activity (Sun et al. 2013). The conserved ZnF motif is critical for DNA binding, enzymatic activity, and downstream signaling activation. Engagement of DNA by cGAS induces rearrangement of the cGAS catalytic pocket to promote binding of the substrates adenosine triphosphate (ATP) and guanosine triphosphate (GTP) to cGAS, resulting in the synthesis of 2′3′ cyclic GMP-AMP (cGAMP) (Civril et al. 2013) (Fig. 1). cGAMP is the ligand of STING, which is an adaptor protein of cGAS and is located on the endoplasmic reticulum (ER) membrane. STING contains a short cytosolic N-terminal fragment, four-span transmembrane helices, a cytosolic ligand-binding domain (LBD), and a C-terminal tail (CTT) (Huang et al. 2012). In the resting state, STING exists as a dimer, and cGAMP binding induces extensive conformational rearrangements to initiate STING oligomerization, activating STING as an effector. STING then leaves the ER membrane, passing through the ER–Golgi intermediate compartment (ERGIC) to translocate to the Golgi (Shang et al. 2019). Trafficking of STING from the ER to the Golgi is mediated by coatomer protein complex II (COPII) vesicles, with the assistance of other proteins, such as GTPase SAR1A, SEC24C, and the ARF-GTPase ARF1 (Gui et al. 2019). At the Golgi membrane, STING recruits TANK-binding kinase 1 (TBK1) and IKK. TBK1 phosphorylates the Ser366 residue in the CTT domain of STING and further recruits IRF3, resulting in the phosphorylation, dimerization, and nuclear translocation of IRF3 (Zhao et al. 2019). In addition, activation of STING leads to canonical NF-κB activation. The activation of IRF3 and NF-kB culminates in target gene expression, including type I interferons and ISGs as well as inflammatory cytokines and chemokines such as IL-6 and IL-12 (Tse and Takeuchi 2023) (Fig. 1).

## The roles of cGAS/STING activation in the development of diseases

### Autoimmune and autoinflammatory diseases

Because the cGAS/STING pathway is activated by self-nucleic acids, the involvement of cGAS/STING activation in the pathogenesis of autoimmune and autoinflammatory diseases has drawn significant attention (Table 1).

**Table 1:** Diseases associated with cGAS/STING pathway

Disease	Relevance with the cGAS/STING signaling	References
*Autoimmune and autoinflammatory diseases*		
Aicardi–Goutières syndrome (AGS)	Inhibition of AGS development in cGAS or STING knockout mice	Gray et al. (2015)
COPA syndrome	Reduction of type I IFN-mediated inflammation by STING deletion or pharmacological inhibition in a mouse model of COPA syndrome (CopaE241K/ +)	Deng et al. (2020)
Familial chilblain lupus	Ligand-independent homodimerization of STING and constitutive expression of type I IFN by heterozygous gain-of-function mutations in STING from familial lupus A patients	König et al. (2017)
STING-associated vasculopathy with onset in infancy (SAVI)	Three mutations in exon 5 of TMEM173 (V147L, N154S and V155M) from SAVI patients Induction of cytokine production, skin ulcerations, lung disease, and premature death by SAVI-mediated STING activation in of STING mutant knock-in mice	Liu et al. (2014), Warner et al. (2017), Bouis et al. (2019), Motwani et al. (2019), Siedel et al. (2020)
Rheumatoid arthritis (RA)	Promotion of inflammatory cytokine production in a cGAS-dependent manner in fibroblast-like synoviocytes from RA patients Reduction of migration and invasion in primary fibroblast-like synovial cells from RA patients by knockdown of cGAS or STING using siRNA in vitro Reduction of inflammatory cell infiltration and joint swelling by cGAS-deficient in an inflammatory arthritis mouse model	Wang et al. (2015b, 2019a), Li et al. (2022a), Willemsen et al. (2021)
Systemic lupus erythematosus (SLE)	In SLE patients, increase of cGAS levels in peripheral blood mononuclear cells (PBMCs) and induction of IFN-I and ISG by activation of cGAS/STING pathway in serum Exacerbation of autoimmunity by lack of cGAS and STING in 2,6,10,14-Tetramethylpentadecane (TMPD) induced chronic SLE mice	An et al. (2017), Kato et al. (2018b), Motwani et al. (2021)
*Cancer*		
Colorectal cancer	Inhibition of inflammation in tumor by recruitment of myeloid cells through STING/type I IFN pathway in mice	Liang et al. (2017)
Gastric cancer	Reduction of STING expression in tumor tissue of gastric cancer patients Positive correlation between STING expression and survival rates of gastric cancer patient	Song et al. (2017)
Hepatocellular Carcinoma (HCC)	Increase of tumor size in STING-deficient HCC mice Impairment of immune surveillance of oncogenic RAS by deletion of STING in mice	Thomsen et al. (2020), Dou et al. (2017)
Lung cancer	Reduction of STING expression in tissues of NSCLC patients with enriched KRAS mutational status	Kitajima et al. (2019)
Prostate cancer	Stimulation of STING-dependent type I IFN expression through accumulation of cytoplasmic DNA by MUS81 in prostate cancer cells	Ho et al. (2016)
Skin cancer	Identification of low STING expression in six melanoma cell lines (MeWo, G361, WM115, SK-MEL-2, SK-MEL-5, and SK-MEL-28) Reduction of B16F10 growth and number of lung metastases by intratumoral injection of 2′,3′-cGAMP in mice Decrease of tumor size and increase of T-cell infiltration by injection of STINGVAX in B16 transplanted melanoma	Xia et al. (2016b), Demaria et al. (2015) Fu et al. (2015)
Metastasis	Promotion of brain metastasis of breast and lung cancer cells by STING activation via the STAT1 and NF-κB pathways in astrocytes Inhibition of lung metastasis by cGAMP via the EMT process and the PI3K/AKT pathway in skin tumor-bearing mice	Bakhoum et al. (2018), Lu et al. (2023)
*Cardiovascular diseases*		
Atherosclerosis	Promotion of initiation and development of atherosclerosis by activated cGAS in ApoE knockout mice	Lu et al. (2021)
Cardiac hypertrophy	Increase of STING expression in cardiomyopathy patients (DCM, HCM) and mice cardiac hypertrophy induced by aortic banding surgery Reduction of cardiac cross-sectional area and inflammatory response by deletion of STING in mouse model of cardiac hypertrophy Reduction of inflammatory response and cardiac hypertrophy by overexpression of STING in mouse model of cardiac hypertrophy	Zhang et al. (2020), Xiong et al. (2021)
Traumatic brain injury (TBI)	Increase of STING mRNA levels in both post-mortem human TBI and mice brain undergoing controlled cortical impact surgery	Abdullah et al. (2018)
Ischemic stroke	In the mouse model of middle cerebral artery occlusion (MCAO), activation of cGAS/STING pathway by accumulation of cytoplasmic dsDNA in microglia and astrocytes Activation of cGAS/STING pathway by the release of mtDNA in the cytoplasm of microglia during ischemic stroke in mice Reduction of brain injury such as brain infarction and brain edema by inhibition of STING in mice Alleviation of ischemic stroke through suppression of microglial M1 polarization by intraperitoneal injection of STING inhibitor in MCAO mice	Li et al. (2020b), Kong et al. (2022)
Myocardial infarction (MI)	Promotion of cardiac repair through increased collagen deposition, fibrogenesis and angiogenesis by silence of cGAS in mice	Cao et al. (2018)
*Kidney diseases*		
Acute kidney injury (AKI)	Induction of cGAS/STING pathway-meditated inflammatory response by cisplatin-induced releasement of mtDNA into the cytoplasm in HK-2 cells Attenuation of AKI in STING knockout mice	Maekawa et al. (2019)
Diabetic kidney disease (DKD)	Increase of STING levels in kidney tissues from DKD mice Increase of cGAS, STING and phosphorylated TBK1 levels in podocytes of db/db mice Induction of podocyte injury by mtDNA-mediated cGAS/STING activation in DKD or diet-induced obesity mice Alleviation of podocyte injury through genetic ablation of STING or pharmacological inhibition by C176 in db/db mice	Khedr et al. (2020), Zang et al. (2022), Mitrofanova et al. (2022a)
Chronic kidney disease (CKD)	Attenuation of renal fibrosis by deletion of STING in mice Increased plaque vulnerability by cGAS-induced type I IFN production due to mitochondrial DNA leakage in mice	Chung et al. (2019), Bi et al. (2021)
*Lung diseases*		
Asthma	Alleviation of ovalbumin- or house dust mite-induced allergic airway inflammation in airway epithelial cell specific cGAS knockout mice	Han et al. (2020)
Chronic obstructive pulmonary disease (COPD)	In bronchoalveolar lavage fluid of mice, Releasement of mitochondrial DNA into the cytoplasm by exposure of cigarette smoke extracts	Pouwels et al. (2016)
Idiopathic pulmonary fibrosis (IPF)	Increase of cGAS and STING expression in lung epithelial cells from IPF patients Reduction of senescence markers by pharmacological cGAS inhibition by RU.521 in airway epithelial cells of IPF Increase of cGAS, STING and self-dsDNA levels in lung tissues of bleomycin-induced fibrosis mouse model Aggravation of lung fibrosis through higher collagen deposition and excessive expression of remodeling factors by deletion of STING in mice Induction of CD8^+^ T cells and chronic activation of type I interferon signaling and immunoproteasome in alveolar epithelial cells of IPF patients via the cGAS/STING pathway	Schuliga et al. (2021), Savigny et al. (2020), Wang et al. (2023a)
Silicosis	Increase of cGAS and STING expression by releasement of self-dsDNA by exposure of silica microparticles in mouse lungs Induction of apoptosis through silica induced STING activation in bone marrow-derived dendritic cells from mice	(Benmerzoug et al. (2018)
*Metabolic disorders and non-alcoholic fatty liver disease*		
High-fat diet (HFD)	Activation of cGAS/STING signaling in adipocytes and iWAT of obese mice fed a HFD	(Bai et al. (2017)
Non-alcoholic fattey liver disease (NAFLD)	Enhance of STING expression in liver tissues from NAFLD patients Induction of glucose and lipid metabolism disorders by promoting liver inflammation and hepatocyte death through the cGAS/STING pathway in HFD mouse model	(Luo et al. (2018) (Qiao et al. (2018)

#### Aicardi–Goutières syndrome (AGS)

Aicardi–Goutières syndrome (AGS) is a rare genetic disease characterized by early onset progressive encephalitis with severe neurological disability and skin lesions. AGS is characterized by high levels of interferon α in cerebrospinal fluid and is considered a type I interferonopathy. AGS is associated with mutations in TREX1, RNASEH2A, RNASEH2B, RNASEH2C, SAMHD1, ADAR1, and IFIH1, all of which encode proteins that function in the detection and metabolism of nucleic acids. TREX1 (three prime repair exonuclease 1) is a DNA 3′ end repair exonuclease that participates in the repair of damaged DNA and the removal of cytosolic DNA. Mutations in the TREX1 gene result in the accumulation of cytosolic DNA and the release of damaged DNA, inducing the constitutive activation of the cGAS/STING pathway and consequently aberrant inflammation and autoimmunity (Gray et al. 2015). Knockout of cGAS or STING protects against the development of AGS in mice with mutations in AGS-related genes (Gray et al. 2015).

#### COPA syndrome

COPA syndrome is a rare early-onset autosomal dominant disease characterized by arthritis, interstitial lung disease, and renal disease and exhibits immune dysregulation and high serum levels of type I interferon (Vece et al. 2016). COPA syndrome is associated with missense mutations in the coatomer-associated protein subunit alpha (COPα) gene. COPα is a subunit of coatomer protein complex I (COPI) and mediates the retrieval of proteins from the Golgi to the endoplasmic reticulum (ER). COPα mutations are linked with ligand-independent activation of STING signaling. Genetic deficiency or pharmacological inhibition of STING mitigated type I interferon-mediated inflammation in a mouse model of COPA syndrome (CopaE241K/ +) (Deng et al. 2020).

#### Familial chilblain lupus

**Fig. 2:**
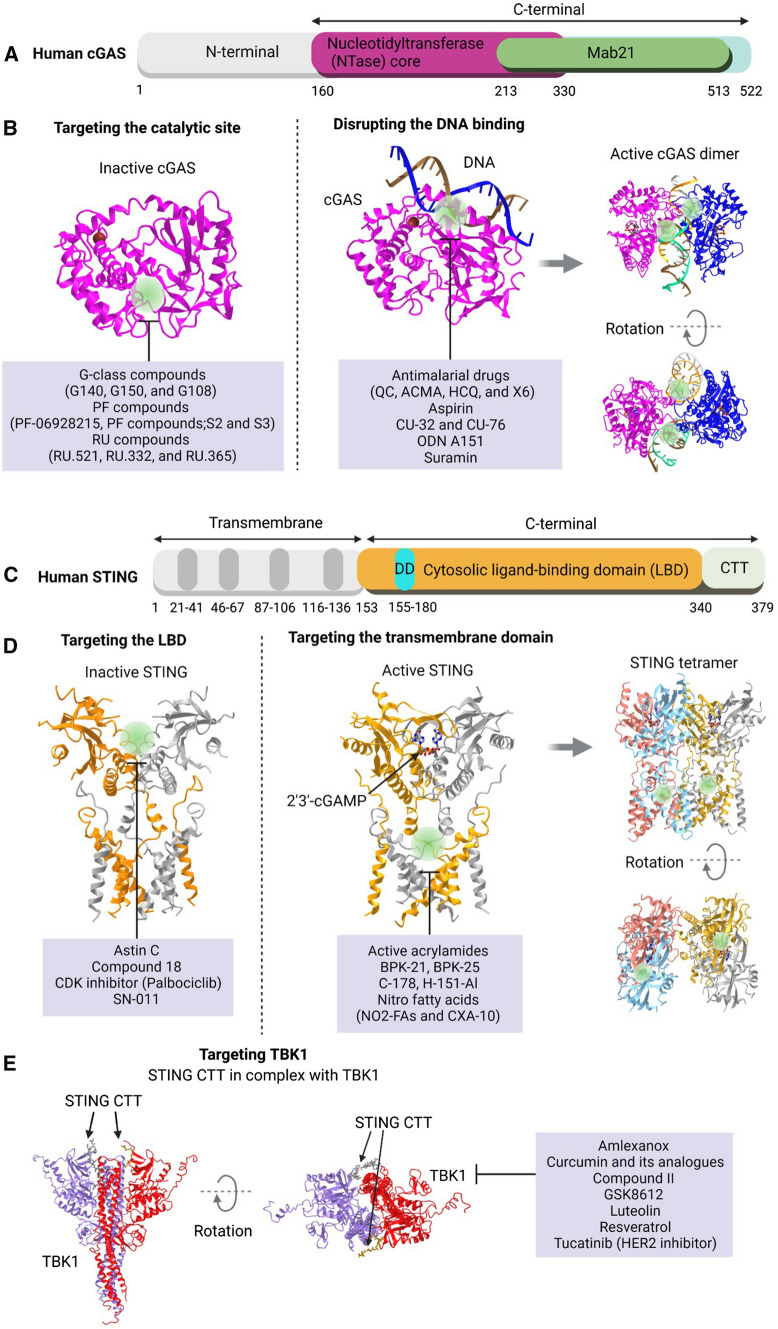
Mechanism of inhibitors targeting cGAS, STING and TBK1. **A** A diagram illustrating the organization of human cGAS domains. **B** Representative inhibitory targets of human cyclic GMP–AMP synthase (cGAS) are shown in three-dimensional structure. Molecules that interfere with the catalytic site and molecules that have been reported to interfere with DNA are each labeled at the target site. The structure shown is modeled as a cGAS catalytic domain (*Homo sapiens* PDB: 4O68) (Li et al. 2013), cGAS DNA binding domain (*H. sapiens* PDB: 6CT9) (Zhou et al. 2018), and a dimer (PDB: 4LEZ). **C** A diagram illustrating the organization of domains in human STING. **D** Representative inhibitory targets of stimulator of interferon genes (STING) are shown in three-dimensional structure. The molecules targeted at the ligand-binding domain and the transmembrane domain, which are the target sites for inhibition, are indicated. The structure shown was modeled as a ligand-binding domain (*H. sapiens* PDB: 6NT5), a transmembrane domain (*H. sapiens* PDB: 6NT7), and a tetramer (*G. gallus* PDB: 6NT8) (Shang et al. 2019). **C** The crystal structure of STING CTT in complex with TBK1 (*H. sapiens* PDB: 6O8C) is shown as a three-dimensional structure, and inhibitors of TBK1 are shown

Familial chilblain lupus is a rare monogenic form of lupus erythematosus characterized by painful cold-induced inflammatory responses on acral surfaces. The disease is known to be associated with a loss-of-function mutation in TREX1 (3′ repair exonuclease 1) or SAMHD1 (Gunther et al. 2015; Linggonegoro et al. 2021) A heterozygous gain-of-function mutation of STING was identified in patients with familial chilblain lupus A (König et al. 2017). A gain-of-function mutation of STING results in ligand-independent homodimerization and constitutive expression of type I IFNs (König et al. 2017). Unmetabolized cytosolic DNA induces activation of the STING pathway to initiate innate immune responses, resulting in higher type I interferon levels that are linked to the pathology of familial chilblain lupus as a type I interferonopathy (Fig. 2).

#### STING-associated vasculopathy with onset in infancy (SAVI)

STING-associated vasculopathy with onset in infancy (SAVI) is an autoinflammatory disease caused by mutations in the STING1 gene, resulting recurrent fevers, ulcerative skin lesions, vasculitis, and interstitial lung disease (Liu et al. 2014). The mutations are on the connector helix loop (N154S, V155M and V147L) and the polymerization interface (G207E, R281Q, R284G and R284S) of STING. The mutations culminate in the ligand-independent activation of STING, inducing spontaneous trafficking of STING to the Golgi to activate downstream signaling pathways and leading to increased type I interferon production. Knock-in of mutations N153S or V154M in mice results in cytokine production, skin ulcerations, lung disease, and premature death (Warner et al. 2017; Bouis et al. 2019; Motwani et al. 2019; Siedel et al. 2020). The STING inhibitor SN-011, which prevents spontaneous STING oligomerization and activation, prohibits type I IFN production and inflammatory gene expression induced by SAVI-associated STING mutations (Hong et al. 2021).

#### Rheumatoid arthritis

dsDNA levels are increased in synovial tissues and fibroblast-like synoviocytes (FLSs) obtained from rheumatoid arthritis (RA) patients (Wang et al. 2015b, 2019a). cGAS expression was also enhanced in RA FLS compared with osteoarthritis FLS and healthy control FLS, with a positive correlation between cGAS expression in tissue and synovitis scores, suggesting an association between cGAS expression and rheumatoid arthritis synovial inflammation (Wang et al. 2019a). In addition, dsDNA increased the expression of inflammatory cytokines such as IL-1β, TNF-α, MMP-13, CXCL-10, IL-6, IL-8, IFN-α, IFN-β and IFN-γ in FLSs of RA patients in a cGAS-dependent manner (Wang et al. 2019a).cGAS or STING is responsible for inflammatory arthritis in *DNase* knockout mice (Ahn et al. 2012; Gao et al. 2015). Loss of DNase II activity results in the accumulation of host DNA in the cytosol, which subsequently triggers inflammatory polyarthritis with the production of inflammatory cytokines such as TNF-α, IL-1β, and IL-6. Polyarthritis symptoms develop in *DNaseII*-/-*Ifnar1*-/- mice but did not appear in *DNaseII*-/-*cGas*-/- mice, suggesting that cGAS is important in DNA-induced arthritis pathology (Gao et al. 2015). DNA-induced production of TNF-α, IL-1β, and IL-6 was abolished in STING-knockout macrophages. In addition, knockout of STING prevents polyarthritis symptoms in *DNaseII*-/- mice, suggesting that self-DNA-induced inflammation and arthritis symptoms are dependent on the activation of STING (Ahn et al. 2012). siRNA knockdown of cGAS or STING reduced cytosolic dsDNA-induced migration and invasion of fibroblast-like synoviocytes (FLSs) obtained from rheumatoid arthritis (RA) patients with diminished formation of lamellipodia (Li et al. 2022a). Furthermore, transfection of RA FLSs with *cGAS* or *STING* shRNA resulted in a decreased capability of FLS invasion into cartilage in the SCID mouse coimplantation model (Li et al. 2022a). Inflammatory cell infiltration and joint swelling are decreased in cGAS-deficient mice in an inflammatory arthritis animal model (Willemsen et al. 2021).

#### Systemic lupus erythematosus (SLE)

Systemic lupus erythematosus is a chronic systemic autoimmune disease that affects the skin, blood, kidney, and musculoskeletal organs (Elbourne et al. 1998). cGAS expression levels were increased in peripheral blood mononuclear cells (PBMCs) from SLE patients compared with PBMCs from normal controls (An et al. 2017). The severity of disease was correlated with cGAMP levels in SLE patients (An et al. 2017). SLE serum collected from SLE patients exerted IFN-I and ISG-inducing activity, at least partly mediated through the activation of the cGAS–STING pathway by elevated dsDNA in SLE serum (Kato et al. 2018b). Activation of STING participated in the development of lupus in Fcgr2b-deficient mice by promoting the maturation and differentiation of dendritic cells, whereas inhibition of STING signaling protected against lupus development (Thim-Uam et al. 2020).

In contrast, a recent study demonstrated that STING and cGAS deficiencies exacerbate disease symptoms in a chronic model of 2,6,10,14-tetramethylpentadecane (TMPD)-induced autoimmunity (Motwani et al. 2021). TMPD-induced aggravation of SLE in cGAS- or STING-deficient mice is dependent on endosomal TLRs. Therefore, the role of the cGAS/STING pathway in promoting autoinflammation does not necessarily translate into SLE development (Motwani et al. 2021). These data provide caveats for the use of cGAS-STING-targeted therapy for SLE treatment.

### Cancer

Unlike normal cells, cancer cells are rich in self DNA and cytoplasmic dsDNA, a byproduct of genomic instability, which activate the cGAS/STING pathway to promote or inhibit tumor development (Woo et al. 2014; Dou et al. 2017; Harding et al. 2017; Mackenzie et al. 2017). Chromosomal abnormalities occur in cancer cells with unstable genomes due to errors during mitosis, which also leads to micronuclei formation (Crasta et al. 2012). Micronuclear envelopes are prone to rupture, and genomic content is readily exposed in the cytoplasm, leading to DNA sensing by cGAS (Mackenzie et al. 2017). Recent advances in the understanding of the mechanisms of the cGAS/STING pathway have played a major role in advancing and improving cancer immunotherapy. The cGAS/STING pathway functions primarily as a tumor suppressor with respect to interferon (IFN) production and T-cell priming. There is also emerging evidence that chronically activated cGAS/STING signaling can induce an immunosuppressive tumor microenvironment. Conversely, studies have also demonstrated that cGAS/STING signaling can promote tumor expression and metastasis under certain circumstances (Ahn et al. 2014; Bakhoum et al. 2018).

#### Colorectal cancer

In a study with human colon cancer cell lines, Xia et al. (2016a, b) revealed that the dsDNA-induced STING signaling pathway was impaired in most of these cell lines and that cGAS and STING expression was insufficient. Furthermore, TBK1 and IRF3 phosphorylation/translocation was rarely observed in some human colon cancer cell lines. This study indicated that the STING pathway functions to suppress intestinal tumorigenesis and that this function can be selectively inhibited during cancer development. Given the importance of the cGAS/STING signal as the host’s defense mechanism against viral infections, colorectal carcinomas exhibiting defective cGAS/STING signaling may be more susceptible to various infections in addition to the oncolytic activity of DNA viruses such as herpes simplex virus (HSV) (Xia et al. 2016a). Another study revealed that STING can regulate the cell cycle in a cGAS-independent manner in certain tumor models, such as HCT116 colorectal carcinoma (Ranoa et al. 2019). These findings are important because they suggest that tumors without cGAS expression can maintain active STING through other DNA sensors. Notably, the potential role of STING in promoting tumor growth and immune evasion is reflected in its high expression in colorectal cancer patients with poor prognoses (An et al. 2019). STING signaling in colorectal adenocarcinoma cells was found to be regulated by HER2 recruitment of AKT1 in a process that disrupted STING signaling and resulted in the inhibition of antiviral defense and the suppression of antitumor immunity (Wu et al. 2019). A recent advance to increase radiotherapy suggested that radiation-induced STING activation acts as an immunosuppressant, which results in M-MDSC infiltration and tumor radiotherapy resistance. The STING/type I interferon pathway suppresses inflammation in tumors in part by recruiting myeloid cells through the CCR2 pathway (Liang et al. 2017). Therefore, treatment with an anti-CCR2 antibody alleviates immunosuppression after activation of the STING pathway, thereby enhancing the antitumor effect of the STING agent and radiotherapy (Liang et al. 2017).

#### Gastric cancer

Chronic *Helicobacter pylori* infection is identified as one of the strongest risk factors for gastric cancer. The function of STING signaling in gastric cancer development was investigated by detecting STING expression in 217 gastric cancer patients who underwent surgical resection (Song et al. 2017). STING protein expression was significantly lower in these tumor tissues than in nontumor tissues, and low STING staining intensity was positively correlated with depth of tumor invasion, tumor size, lymph node metastasis, decreased patient survival, and tumor, node and metastasis (TNM) stage. Multivariate analysis identified STING as an independent prognostic factor that could improve the predictive accuracy of overall survival when incorporated into the TNM staging system. Chronic *H. pylori* infection upregulated STING expression and activated STING signaling in mice. Although reduced expression of STING in gastric cancer is not directly caused by *H. pylori* infection, it can significantly affect tumorigenesis by inhibiting immune surveillance. In conclusion, STING is proposed as an independent novel prognostic factor and a potential immunotherapeutic target for gastric cancer (Song et al. 2017). MUS81 is known to suppress the chromosomal instability (CIN) that arises from damaged replication forks by cleaving potentially harmful DNA structures (Ciccia et al. 2008). In a recent study, MUS81 inhibition enhanced the sensitivity of the anticancer effect of the WEE1 inhibitor MK1775 in gastric cancer in vitro and in vivo. Here, MUS81 inhibition increased the accumulation of cytoplasmic DNA induced by MK1775 treatment and activated the DNA sensor STING-mediated innate immunity in gastric cancer cells. Therefore, MK1775 can potentiate the anticancer effect of immune checkpoint blockade therapy by activating cGAS/STING signaling, especially in MUS81-deficient gastric cancer cells (Li et al. 2021).

#### Hepatocellular carcinoma

Hepatocellular carcinoma (HCC) is the most common primary liver cancer. Thomsen et al. (2020) explored the therapeutic efficacy of targeting the DNA-activated STING pathway in HCC using a mutagenic HCC mouse model. STING-deficient mice possessed more large tumors during the later stages of HCC. The levels of phospho-STAT1, autophagy, and cleaved caspase-3 were reduced in the livers of STING-deficient HCC mice. These changes were restored in the liver by treatment with a cyclic dinucleotide (CDN) STING agonist, and the tumor size was effectively reduced. Overall, modulation of the STING pathway influences HCC progression; thus, STING agonist treatment could be used in combination with other immunomodulatory therapies or standard therapies, such as PD1 inhibitors, against HCC (Thomsen et al. 2020). Dou et al. (2017) induced immune-mediated elimination of precancerous hepatocytes by activating RAS-induced hepatocyte senescence, senescence-associated secretory phenotype (SASP), and inflammation. This study confirmed that STING-deficient mice exhibit impaired immune surveillance of oncogenic RAS, which can lead to malignancy (Dou et al. 2017). Qi et al. (2020) studied the prognostic value and correlation of the cGAS/STING pathway with immune infiltration based on database analysis in HCC. Their results demonstrate that potential kinase targets in the cGAS/STING pathway include the SRC family of tyrosine kinases, phosphoinositide 3-kinase-related protein kinase family kinases, and mitogen-activated protein kinase 1. A significant correlation in HCC was also confirmed between the expression of the cGAS/STING pathway and the infiltration of various immune cell types, including B cells, CD4^+^ T cells, CD8^+^ T cells, macrophages, dendritic cells, and neutrophils. Expression of the cGAS/STING pathway also exhibited a strong relationship with a diverse set of immune markers in HCC. Persistent DNA damage caused by defective breast cancer gene (BRCA) pathway (disrupted BRCA1-PALB2 interaction) induces tumor immunosuppression through the cGAS-STING pathway, while also promoting T-lymphocyte infiltration. This finding provides important insights into the reconfiguration of the tumor immune microenvironment, which is helpful in enhancing the response to PD-1 antibody treatment for HCC (Ma et al. 2023). These results suggest that members of the cGAS/STING pathway can be used as prognostic biomarkers and that immunotherapy can be targeted in HCC patients (Qi et al. 2020).

#### Lung cancer

KRAS-LKB1 (KL)-mutant lung cancers are particularly aggressive, lack PD-L1 expression and do not respond positively to immune checkpoint blockades (ICBs) (Skoulidis et al. 2018). Kitajima et al. (2019) reported that LKB1 loss resulted in marked silencing of STING expression and insensitivity to cytoplasmic dsDNA sensing. Suppression of STING expression results from a combination of hyperactive DNMT1 and EZH2 with selection pressure to avoid the deleterious effects of mitochondrial stress and cytosolic mtDNA release*.* Therefore, low levels of tumor cell STING expression are a promising general biomarker for intrinsic resistance to ICB (Kitajima et al. 2019). 5,6-dimethylxanthenone-4-acetic acid (DMXAA), the first agonist targeting the STING pathway, was initially used as an antiangiogenic drug. However, treatment with DMXAA failed to yield significant benefits in phase 3 trials with non-small cell lung cancer patients because DMXAA does not bind human STING despite being a competitive mSTING agonist with a strong affinity (Lara et al. 2011).

#### Prostate cancer

Ho et al. (2016) reported that dsDNA is present in the cytosol of human prostate carcinoma DU145 cells, human adenocarcinoma PC-3 cells, and the mouse prostate tumor cell line TRAMP-C2, which was derived from spontaneously developing prostate tumors in transgenic TRAMP mice. MUS81 inhibits chromosomal instability (CIN) resulting from disrupted replication by cleaving potentially harmful DNA structures. Cleavage of genomic DNA by the DNA structure-specific endonuclease MUS81 and the PARP-dependent DNA repair pathway induces the accumulation of cytoplasmic DNA in prostate cancer cells. Both the number of nuclear MUS81 foci and the amount of cytoplasmic dsDNA increased in parallel from hyperplasia to clinical stage II prostate cancer and decreased in stage III. Cytoplasmic DNA produced by MUS81 stimulates DNA sensor STING-dependent type I IFN expression and promotes phagocytic and T-cell responses that result in type I and II IFN-mediated prostate tumor cell rejection through a partially macrophage-dependent mechanism. The results reveal that the tumor suppressor MUS81 notifies the immune system of the presence of transformed host cells (Ho et al. 2016).

#### Skin cancer

STING deficiency has been associated with skin cancer incidence. STING expression was undetectable or significantly suppressed in six melanoma cell lines (MeWo, G361, WM115, SK-MEL-2, SK-MEL-5, and SK-MEL-28) (Xia et al. 2016b). Demaria et al. (2015) demonstrated that intratumoral injection of 2′,3′-cGAMP significantly delayed tumor growth in a B16F10 mouse model. This study also demonstrated that intratumoral injection of GAMPs potentiates the anticancer CD8^+^ T-cell response, a property that can be further enhanced when both PD-1 and CTLA-4 are blocked. The authors further report that this immune response was dependent on the production of type I IFN from endothelial cells in the tumor microenvironment, indicating the potential of a strategy targeting tumor endothelial cells for melanoma immunotherapy (Demaria et al. 2015). In another study, injection of STINGVAX into the contralateral segment of B16-transplanted melanoma significantly suppressed tumor size and increased T-cell infiltration in the tumor tissue in a dose-dependent manner (Fu et al. 2015). Cyclic diguanylate monophosphate (c-di-GMP), which activates STING, enhances the immunogenic and antitumor effects of a peptide vaccine against mouse B16 melanoma (TriVax boost immunization using the hgp100 peptide epitope (KVPRNDQWL))(Wang and Celis 2015). Reduced and delayed tumor growth was also observed in a B16 melanoma mouse model treated with a combination of CDN-based poly β-amino ester (PBAE-CDN) nanoparticles and anti-PD-1 therapy (Wilson et al. 2018). Talimogene laherparepvec, an oncolytic immunotherapy, was demonstrated to be effective in treating patients with advanced melanoma in a phase 3 clinical trial (Andtbacka et al. 2015). In their study, STING-deficient melanoma cells were susceptible to viral infection, whereas cancer cells whose STING pathway remained intact grew rapidly. Given that STING deficiency alongside oncolytic virus treatment is associated with improved prognosis, further in vivo experiments and clinical trials will allow us to develop prognostic and predictive biomarkers for oncolytic immunotherapy for cancer patients. However, we cannot ignore the fact that chronic stimulation of the cGAS/STING pathway can lead to inflammation-induced carcinogenesis. Ahn et al. (2014) found that mutagenic 7,12-dimethylbenz(a)anthracene (DMBA), cisplatin, and etoposide induced nuclear DNA leakage into the cytoplasm, activating the production of STING-dependent cytokines. Notably, bone marrow transplant experiments suggest that STING in hematopoietic stem cells plays a critical role in DMBA-induced skin tumorigenesis (Ahn et al. 2014).

#### Metastases

The cGAS/STING pathway has been shown to promote brain metastasis. STING activation in astrocytes mediates brain metastasis of breast and lung cancer cells. Interestingly, cGAMP produced by cancer cells translocates across the carcinoma-astrocytic gap junction and activates STING in astrocytes. In response to STING activation, inflammatory cytokines and tumor necrosis factors are produced, which activate the STAT1 and NF-κB pathways in cancer cells. These paracrine effects promote cancer cell growth and confer chemoresistance to metastatic brain cells (Chen et al. 2016). Another study provided a correlation between cGAS/STING activation and human brain cell metastasis. Here, CIN formed by chromosome mis-segregation during cell division promoted micronucleus formation and activated the cGAS/STING pathway to induce noncanonical NF-κB signaling in metastasis models but not type I IFN signaling. CIN-induced metastasis depends on both STING and NF-κB signaling and is associated with epithelial-to-mesenchymal transition and the induction of inflammation-related genes (Bakhoum et al. 2018). The cGAS/STING pathway is known to inhibit lung metastasis. Lung metastases were induced by the intravenous injection of B16F10 tumor cells into cGAMP-injected skin tumor-bearing mice. After 10 days, the number of melanoma metastases in their lungs was counted. Intratumoral injection of cGAMP strongly reduced the number of lung metastases, suggesting that systemic immunity was induced to suppress metastasis formation. The STING agonist 2′,3′-cGAMP has been shown to activate the cGAS-STING-IRF3 pathway and modify the tumor immune microenvironment in the treatment of solid tumors (triple-negative breast cancer (TNBC) cells). It prevented tumor metastasis by reversing the EMT (Epithelial-Mesenchymal Transition) process and the PI3K/AKT pathway (Lu et al. 2023).

In summary, intratumoral cGAMP treatment is effective in the growth retardation of injected tumor cells as well as in contralateral tumors (Demaria et al. 2015). It is essential to develop a comprehensive understanding of the activation of the cGAS/STING pathway, which may possess both antitumor and protumor roles depending on the cancer type and stage of cancer progression. As summarized and discussed, activation of the cGAS/STING pathway plays important roles throughout the entire process of tumorigenesis to cancer metastasis. Therefore, cGAS and STING are potential biomarkers to improve the chemotherapy prognosis and effectively prevent immune evasion of tumors.

### Cardiovascular diseases

Atherosclerosis is the narrowing of arteries due to the accumulation of plaques, which are composed of smooth muscle cells, macrophages, lipids, and cholesterols. In a study on atherosclerosis, the induction of the inflammatory response by activation of cGAS promoted the initiation and development of atherosclerosis (Lu et al. 2021). Analysis of the Gene Expression Omnibus dataset revealed that cGAS expression in the aorta and macrophages of apolipoprotein E knockout mice (ApoE^−/−^) was higher than that in wild-type (WT) mice. Furthermore, inhibition of cGAS in RAW264.7 cells suppressed lipopolysaccharide-mediated M1 polarization and decreased the mRNA levels of proinflammatory cytokines (IL-1β and IL-7) and genes involves in cholesterol uptake (CD36 and MSR1) and cholesterol esterification and hydrolysis (ACAT1 and ACAT2) (Lu et al. 2021).

Cardiac remodeling, including cardiac hypertrophy, is a major progressive cause of chronic heart failure (CHF). The expression of STING is increased in patients with dilated cardiomyopathy (DCM) and hypertrophic cardiomyopathy (HCM) (Zhang et al. 2020). The mouse model of cardiac hypertrophy demonstrated reduced cardiac cross-sectional area and inflammatory response due to STING deletion, which also inhibited phosphorylation of ER stress markers, including protein kinase RNA (PKR)-like ER kinase (PERK), inositol-requiring enzyme 1α (IRE-1α), and eukaryotic translation initiation factor 2α (eIF2α). STING knockdown in neonatal rat cardiomyocytes (NRCMs) reduced mRNA levels of cardiac hypertrophy markers, such as atrial natriuretic peptide (ANP) and B-type natriuretic peptide (BNP), which were increased by angiotensin-II (Ang-II) treatment (Zhang et al. 2020). Conversely, overexpression of STING reduced the cardiac inflammatory response and inhibited cardiac hypertrophy (Xiong et al. 2021). In the mouse model of cardiac hypertrophy, overexpression of STING resulted in smaller myocardial cell size and reduced cardiac fibrosis compared with WT mouse hearts and improved cardiac function, including cardiac ejection fraction. Overexpression of STING in the heart inhibited autophagy by reducing the levels of autophagy-related proteins, including Beclin-1, Atg7, and Atg12 (Xiong et al. 2021). Owing to the conflicting results of Zhang et al. (2020) and Xiong et al. (2021), the role of STING in cardiac hypertrophy requires further investigation.

Traumatic brain injury (TBI) is a chronic neuroinflammatory response due to continuous damage to nerve cells, resulting in their death as a secondary response. The mRNA level of STING was increased in both postmortem human brain tissue and brain tissue from a mouse model of TBI (Abdullah et al. 2018). STING expression was localized in astrocytes and neurons in the mouse model of TBI. In TBI, deletion of STING reduced lesion volume of the brain and reduced levels of inflammatory cytokines, including TNF-α and IL-1β, compared with those in WT mice (Abdullah et al. 2018).

Ischemic stroke is a serious neurological disease caused by irreversible brain damage due to initial ischemia and inflammation following ischemia. In a study on ischemic stroke, the inhibition of cGAS improved ischemic brain injury (Li et al. 2020b). In a mouse model of middle cerebral artery occlusion (MCAO), dsDNA accumulated in the cytoplasm of microglia and astrocytes, and accumulated dsDNA caused activation of cGAS/STING signaling. Furthermore, the activation of cGAS/STING signaling induced pyroptosis by increasing the expression of inflammatory cytokines, including caspase-1 and IL-1β in microglia and astrocytes. Intraperitoneal injection of A151, a cGAS antagonist, reduced the expression of inflammatory cytokines and prevented microglial pyroptosis in the brains of MCAO mice (Li et al. 2020b). During ischemic stroke, mtDNA is released into the cytoplasm, which activates cGAS/STING signaling in microglia (Kong et al. 2022). Moreover, this study demonstrated that the suppression of STING reduced brain injury, including brain infarction and brain edema. Suppression of STING by intraperitoneal injection of C-176, a STING inhibitor, alleviated ischemic stroke in MCAO mice by inhibiting microglial M1 polarization. In BV2 microglial cells, the suppression of STING reduced the expression of M1-related markers, including tumor necrosis factor-α (TNF-α) and inducible nitric oxide synthase (iNOS), and increased that of M2-related markers, including arginase-1 (Arg-1) and IL-10 (Kong et al. 2022).

Myocardial infarction (MI) is accompanied by inflammatory and immune responses and results in massive cardiomyocyte death due to overactive cGAS/STING signaling. Wang et al. (2015a, b) demonstrated that mtDNA levels increased in the plasma from patients with acute myocardial infarction (AMI) through qPCR analysis (Wang et al. 2015a). Because cGAS is activated by dsDNA, including mtDNA, increased levels of mtDNA might cause cGAS-mediated inflammatory responses in AMI patients. Cao et al. (2018) demonstrated that the cGAS/STING signaling pathway regulates the transformation of macrophages in the infarct zone and cardiac repair after injury (Cao et al. 2018). Silencing of cGAS has been shown to promote transformation into reparative macrophages and higher collagen deposition during myocardial ischemia in mouse hearts. Moreover, cGAS silencing has been shown to enhance cardiac repair, fibrogenesis and angiogenesis after injury (Cao et al. 2018).

MI in mice induces IRF3 activation in interferon-inducible cells, a type of heart-specific macrophage (King et al. 2017). Disruption of IRF3 reduced inflammatory cytokine and chemokine expression and inflammatory cell infiltration. Furthermore, treatment with an IFNAR-neutralizing antibody after MI alleviated left ventricular dysfunction and improved patient survival. This study suggests that these are potential therapeutic targets for myocardial infarction (King et al. 2017).

### Kidney disease

The cGAS/STING signaling pathway has been shown to regulate inflammation and energy homeostasis in acute and chronic renal disorders (Mitrofanova et al. 2022b). Mitochondrial damage and the subsequent release of mitochondrial DNA into the cytosol culminate in the activation of the cGAS/STING pathway and are therefore considered the major causes of renal injury pathology.

Acute kidney injury (AKI) results from rapid disruption in kidney function, is particularly prevalent in hospitalized patients and is associated with various causes, such as sepsis, cardiac surgery, rhabdomyolysis, and drug toxicity (Beyett et al. 2018). AKI is characterized by excessive inflammation and tubular damage with high morbidity and mortality rates (Zuk and Bonventre 2016). Mitochondrial dysfunction has been identified as an important etiology for tubular cell damage and kidney failure in AKI (Liu et al. 2021). Mitochondrial DNA is released into the cytosol in damaged renal tubular cells and is detected by cGAS, inducing the immune and inflammatory responses mediated by the cGAS/STING pathway (Maekawa et al. 2019). In cisplatin-induced AKI, mitochondrial DNA leakage and the subsequent activation of the cGAS/STING pathway play a critical role in the pathology of cisplatin-induced inflammation (Maekawa et al. 2019). In STING KO mice, cisplatin-induced AKI was significantly attenuated compared with that in WT mice (Maekawa et al. 2019).

Chronic kidney disease, including diabetic kidney disease (DKD), is a major health problem worldwide because there is no effective treatment. STING levels are significantly higher in kidney tissues isolated from DKD animal models of eNOS^db/db^ mice and type 2 diabetic nephropathy (T2DN) rats and type 2 diabetes patients compared with controls (Khedr et al. 2020). Podocyte injury is one of the hallmarks of early-stage damage in the development of DKD, resulting in renal dysfunction in diabetic db/db mice (Zang et al. 2022). The expression of cGAS and STING proteins is increased with enhanced TBKI phosphorylation in glomerular podocytes of 8-week-old db/db mice (Zang et al. 2022). Genetic ablation of STING or pharmacological inhibition by C176 rescued podocyte injury in diabetic db/db mice. Activation of cGAS/STING by cytosolic mtDNA mediates lipotoxicity-induced podocyte injury in DKD or diet-induced obesity (Mitrofanova et al. 2022a; Zang et al. 2022).

Mitochondrial defects, including a loss of mitochondrial transcription factor A (TFAM) in renal tubular cells, were observed in the kidneys of patients and animals with fibrosis (Chung et al. 2019). Mice with tubule-specific TFAM deficiency (Ksp-Cre/Tfamflox/flox) exhibited severe mitochondrial loss, kidney fibrosis, and immune cell infiltration (Chung et al. 2019). Translocation of mitochondrial DNA (mtDNA) to the cytosol occurs in renal cells, inducing activation of the cGAS/STING pathway and immune cytokine expression (Chung et al. 2019). Deletion of STING attenuated symptoms of renal fibrosis, indicating the critical role of cGAS/STING in the pathology of renal fibrosis (Chung et al. 2019).

Chronic kidney disease (CKD)-induced oxidative stress culminates in mitochondrial damage to trigger cGAS-STING activation and IFN production in vascular smooth muscle cells (VSMCs), increasing atherosclerotic plaque vulnerability (Bi et al. 2021). These results demonstrate that the promotion of CKD-associated plaque vulnerability is mediated by cGAS/STING activation in VSMCs (Bi et al. 2021).

### Lung diseases

Self-DNA accumulation in the cytosol is one of the main causes of lung diseases. House dust mites (HDMs) induced DNA double-strand breaks in the bronchial epithelium of an asthma mouse model, which were considered to be linked to airway inflammation and allergic responses (Chan et al. 2016). Cytosolic dsDNA accumulation was observed in airway epithelial cells of ovalbumin- or HDM-induced asthmatic mice (Han et al. 2020). Cell-specific deletion of cGAS in airway epithelial cells markedly alleviated OVA- or HDM-induced allergic airway inflammation, reducing eosinophil cell infiltration and production of Th2 cytokines, GM-CSF, IL-25, and IL-33 (Han et al. 2020). Furthermore, total and HDM-specific serum IgE levels and IgE-positive B-cell fractions in bronchoalveolar lavage fluid and mediastinal lymph nodes were lower in STING knockout mice than in wild-type mice in an HDM-induced allergic asthma model, whereas a STING ligand, cyclic GMP-AMP, increased total and HDM-specific serum IgE levels and B-cell proportions in BALF (Gijon Mancheno et al. 2021). Interestingly, intranasal challenge of mice with cyclic-di-GMP (CDG), which is a bacterial product ubiquitously present as a secondary messenger, induced a shift of ILC2s to ILC1s and suppressed *Alternaria*-induced type 2 inflammation in the lung in a STING-dependent manner (Cavagnero et al. 2021). This response is in contrast to the role of STING in the gastrointestinal mucosa, as the ILC2 population and type 2 cytokines such as IL-4 and IL-13 were downregulated while ILC1 frequency was increased in the gut of STING-deficient mice (Canesso et al. 2018). These results demonstrate that the role of the cGAS/STING pathway in type 2 immune responses should be further explored according to tissue, stimulus type, and context.

Chronic obstructive pulmonary disease (COPD) is a severe inflammatory disease with emphysema and fibrosis. Cigarette smoke exposure is considered a major etiological cause of COPD (Mannino and Buist 2007). Cigarette smoke extract (CSE) exposure to mice induced the release of double-stranded DNA and mitochondrial DNA along with other DAMPs, such as ATP and HMGB1, in the bronchoalveolar lavage fluid of mice (Pouwels et al. 2016). Acute cigarette smoke exposure increased self-DNA levels in the bronchoalveolar space with neutrophil infiltration and pulmonary expression of the cGAS and STING proteins in the lungs (Nascimento et al. 2019). cGAS and STING were required for lung inflammation induced by cigarette smoke exposure (Nascimento et al. 2019).

Idiopathic pulmonary fibrosis (IPF) is a common type of pulmonary fibrosis characterized by scarring (fibrosis), thickening, and stiffening of lung tissue, making it difficult to breathe. The damage from IPF is irreversible and progressive, eventually resulting in respiratory failure (Richeldi et al. 2017). Extracellular mtDNA levels were increased in the bronchoalveolar lavage fluid and the plasma of IPF patients, which is associated with disease progression and reduced survival (Ryu et al. 2017). cGAS and STING expression in lung epithelial cells from IPF patient lungs was higher than that in control donors (Schuliga et al. 2021). Airway epithelial cells (AECs) from IPF patients exhibited high baseline senescence and higher mtDNA release, whereas a pharmacological cGAS inhibitor, RU.521, reduced senescence markers in IPF-AECs (Schuliga et al. 2021). In contrast, Savigny et al. reported that self-DNA levels were elevated and that cGAS and STING expression was increased in lung tissues in a bleomycin-induced fibrosis mouse model, and STING deficiency aggravated the progression of lung fibrosis with higher collagen deposition and excessive expression of remodeling factors (Savigny et al. 2020). Cytoplasmic DNA sensing through the cGAS/STING pathway serves as an activator for the immunoproteasome and CD8^+^ T cells, uncovering a new potential pathological mechanism for pulmonary fibrosis (Wang et al. 2023a).

Intratracheal exposure of mice to silica microparticles induced lung cell death and self-dsDNA release in the bronchoalveolar space with lung inflammation, along with increased expression of cGAS and STING in the lungs (Benmerzoug et al. 2018). DNase I treatment in mice blocked silica-induced STING activation, as shown by STING expression, phosphorylation and dimer formation, and TBK1 and IRF3 phosphorylation in lung homogenates (Benmerzoug et al. 2018). Silicosis patients exhibited increased circulating dsDNA in blood, and patients with fibrotic interstitial lung disease exhibited STING activation, as determined by STING expression, phosphorylation and dimer formation, and TBK1 and IRF3 phosphorylation (Benmerzoug et al. 2018). Silica-induced inflammation and cell death were dependent on the cGAS/STING pathway via detection of self-DNA by cGAS (Benmerzoug et al. 2018).

### Metabolic disorders and nonalcoholic fatty liver disease

The expression of cGAS and STING and activation markers such as phosphorylation of TBK1, NF-κB p65, and IRF3 were increased in inguinal white adipose tissue (iWAT) and adipocytes from iWAT obtained from high-fat diet (HFD)-fed obese mice (Bai et al. 2017). Cytosolic mtDNA accumulation was distinctly observed in iWAT adipocytes from HFD-fed obese mice, suggesting activation of the cGAS/STING pathway by mtDNA in adipocytes of obese mice (Bai et al. 2017). The saturated fatty acid palmitic acid induces activation of the cGAS/STING pathway via mitochondrial damage and the consequent release of mitochondrial DNA into the cytosol in endothelial cells, promoting ICAM-1 expression and endothelial inflammation (Mao et al. 2017). High-fat diet (HFD)-induced ICAM-1 expression in endothelial cells and macrophage infiltration in adipose tissue as well as insulin resistance and glucose intolerance were abrogated in STING-deficient mice (Mao et al. 2017). IRF3 activation is reported to play a role in the regulation of adipocyte inflammation, insulin resistance and glucose metabolism (Kumari et al. 2016). These findings consistently suggest the involvement of cGAS/STING activation in the promotion of obesity and consequent metabolic dysfunction via the dysregulation of mitochondrial homeostasis.

The etiology of nonalcoholic fatty liver disease (NAFLD) is closely linked to obesity and metabolic dysfunction, progressing to nonalcoholic steatohepatitis (NASH) and cirrhosis. The expression level of STING is enhanced in liver tissues from NAFLD patients (Luo et al. 2018). Activation of IRF3 downstream of STING promoted hepatic inflammation and hepatocyte cell death by disrupting glucose and lipid metabolism in a high-fat diet (HFD) mouse model, whereas STING deficiency attenuated hepatic lipid accumulation (Qiao et al. 2018). Similarly, STING deficiency alleviated hepatic steatosis, fibrosis, and inflammation in a methionine- and choline-deficient diet (MCD) model and a high-fat diet (HFD) model, with reduced cholesterol and triglyceride levels in serum, possibly mediated through the leakage of mtDNA into the cytosol (Yu et al. 2019). STING-expressing cells were increased in livers from NASH patients, and the increased frequency was well correlated with the severity of inflammation and fibrosis stage (Wang et al. 2020).

## Therapeutic regulation of cGAS-STING pathways

Inhibitors and activators of cGAS and STING are potential drugs for the treatment of several diseases, such as cancer and autoimmune disorders, respectively. Thus, further understanding of these modulators can provide insights into new therapeutic avenues.

### cGAS inhibitors

Currently, cGAS-targeting inhibitors are divided into two types according to the mode of action: those targeting the catalytic site of cGAS and those interfering with the DNA binding of cGAS (Fig. 2, Table 2).

**Table 2:** Inhibitors of cGAS, STING, and TBK1, and their therapeutic potentials

Target	Mechanism		Inhibitor	Studies	References
cGAS	Catalytic domain		G-class compounds (G140, G150, and G108)	Inhibition of IFN-β mRNA and CXCL10 mRNA in THP1 cells and primary human macrophages	Lama et al. (2019)
			PF compounds (PF-06928215, PF compounds S2, and S3)	In vitro assay for structural studies and the catalytic mechanism of cGAS	Hall et al. (2017)
				In silico screening and enzyme activity assay	Zhao et al. (2020)
			RU compounds (RU.521, RU.332, and RU.365)	Inhibition of IFN expression in macrophages derived from the AGS mouse model	Vincent et al. (2017)
	DNA-binding domain		Antimalarial drugs (AMDs): quinacrine (QC), 9-amino-6-chloro-2-methoxyacridine (ACMA), hydroxychloroquine (HCQ), and X6	In silico screening of chemical and drug libraries	An et al. (2015)
				Inhibition of ISGs in Trex1-deficient mice and PBMCs from SLE patients	An et al. (2018)
			Aspirin	Inhibition of autologous DNA-induced autoimmunity in Aicardi-Goutières syndrome (AGS) patient cells and an AGS mouse model	Dai et al. (2019)
				about 2,200 clinical trials registered on the NIH list (NCT04132791, NCT02804815)	
			CU-32 and CU-76	Selective inhibition of IRF3 activation in THP1 cells	Padilla-Salinas et al. (2020)
			ODN A151	Inhibition of type I IFN production in human monocytes and Trex1-deficient THP-1 cells	Steinhagen et al. (2018)
			Suramin	Inhibition of IFN-β expression in THP1 cells	Wang et al. (2018b)
				about 21 clinical trials registered on the NIH list (NCT04496596)	
	Indirect inhibition	Inhibiton of BAF activity	Brazilin and Obtusilactone B	BAF as a protein that competes with the cGAS component of this pathway for binding to genomic self-DNA	
				Inducing tumor cell death in vitro	Kim et al. (2013b)
				Inducing abnormal nuclear envelope reassembly and cell death	Kim et al. (2015)
		G3BP1 inhibition	Epigallocatechin-3-gallate (EGCG)	G3BP1 as a factor that promotes the formation of cGAS complex and enhances cGAS binding to DNA	
				Inhibition of inflammatory response in AGS mouse model and IFN-stimulated gene expression in cells from AGS patients	Liu et al. (2019)
		Inhibition of cGAS activity	Perillaldehyde (PAH)	Unclear mechanism	
				Reduction of autologous DNA-induced autoinflammatory response in AGS mouse model	Chu et al. (2021)
STING	Direct inhibition	Ligand-binding domain	Astin C	Blocking the recruitment of IRF3 in Trex1—/- BMDM cells of an autoimmune disease model	Li et al. (2018)
			Compound 18	Stabilization of the open conformation of STING	Siu et al. (2019)
			Cyclin-dependent protein kinase (CDK) inhibitor: Palbociclib	Improvement of autoimmune disease features induced in dextran sodium sulfate (DSS) or Trex1-KO mice	Gao et al. (2022)
			SN-011	Inhibition of IFN and inflammatory cytokine induction activated by 2′3′-cGAMP, herpes simplex virus type 1 infection, Trex1 deficiency, overexpression of cGAS-STING, or the SAVI mutation	Hong et al. (2021)
		Transmembrane domain	Active acrylamides, BPK-21 and BPK-25	Reduction of immune-related proteins and cytokine secretion in primary human T cells	Vinogradova et al. (2020)
			C-178, and H-151-Al	Inhibition of STING activity both in human cells and in vivo	Haag et al. (2018)
			Nitro fatty acids (NO2-FAs/CXA-10)	Inhibition of STING palmitoylation and TBK1 phosphorylation in fibroblasts from patients with STING-associated vascular disease (SAVI)	Hansen et al. (2018)
				Clinical trials (NCT03422510) for oral use in the treatment of primary focal segmental glomerulosclerosis (FSGS)	
	Indirect inhibition	AMPK inhibitor	Compound C	Ability to reduce cGAMP accumulation	
				Rescue of the autoimmune phenotype in a mouse model with Trex1 gene deficiency	Lai et al. (2020)
		NRF2 inducers	4-OI or sulforaphane	Reduction of STING-dependent release of type I IFNs from SAVI-derived fibroblasts	Olagnier et al. (2018)
TBK1	Direct inhibition		Amlexanox	Structural analysis for TBK and IKKε inhibitory mechanism	Beyett et al. (2018)
			Curcumin and its analogues	Computer-based study for TBK inhibitors	Ullah et al. (2020)
	Indirect inhibition		Compound II	Reduction of IFN gene signature in patient lymphoblasts with Trex1 mutation	Hasan et al. (2015)
			GSK8612	Inhibition of IFN-β secretion in THP1 cells	Thomson et al. (2019)
			Luteolin	Inhibition of TBK1-kinase activity and IRF3 dimerization and phosphorylation	Lee et al. (2009)
			Resveratrol	Inhibition of TBK1-kinase activity and the NF-κB activation induced by RIP1 in RAW264.7 cells	Youn et al. (2005)
			Tucatinib (HER2 inhibitor)	Recruitment of the downstream protein kinase AKT1 and phosphorylation of TBK1, blocking STING and TBK1 complex formation and triggering ubiquitination of TBK1	Kulukian et al. (2020)
Other			Compound 13	Reduction of CXCL10 mRNA levels after stimulation with dsDNA	Huffman et al. (2020)

#### Catalytic site inhibitors

##### G-class compounds (G140, G150, and G108)

Lama et al. (2019) developed an ATP-coupled high-throughput luminescence-based detection method and screened a library of nearly 300,000 compounds to identify small molecule inhibitors of human cGAS. Only the most potent human cGAS-specific derivatives with added methylpyrazole (G140), 2-amino-pyridine (G150) or pyrazole (G108) moieties exhibited inhibitory activities in THP1 cells and primary human macrophages. Moreover, G140 and G150 did not possess any off-target effects across a variety of sensors, whereas 10 μM G108 inhibited the cGAMP-stimulated STING pathway and the hairpin RNA-stimulated RIG-I pathway by 20–40% in THP1 cells. Therefore, G-class compounds, especially G140 and G150, are promising candidates for human cGAS drug development and have resulted in more potent mouse cGAS inhibitors than the previously identified RU.521 (Lama et al. 2019).

##### PF compounds (PF-06928215, and PF compounds; S2 and S3)

By screening the Pfizer fragment chemistry library, Hall et al. identified several ligands of human cGAS. PF-06928215 efficiently bound to cGAS and exhibited high inhibitory activity in vitro (Hall et al. 2017). Zhao et al. performed a molecular dynamics simulation of PF-06928215 and the crystal structure of the complex catalytic domain of human cGAS through virtual screening. Based on their findings, they conducted virtual screening to discover new scaffolds for human cGAS inhibitors and observed improved efficacies for the h-cGAS inhibitors, compounds S2 (IC_50_ = 13.1 ± 0.09 μM) and S3 (IC_50_ = 4.9 ± 0.26 μM) (Zhao et al. 2020).

##### RU compounds (RU.521, RU.332, and RU.365)

Vincent et al. (2017) identified RU.365 and RU.332 occupying the active site of mouse cGAS by screening 123,306 compounds using a RapidFire mass spectrometry system (RF-MS). Structure-directed chemical synthesis of subsequent analogs identified RU.521, which exhibited good activity in macrophages derived from the AGS mouse model (IC_50_ = 700 nM) (Vincent et al. 2017). Based on the significant inhibitory effects of RU family compounds on murine cGAS, they are expected to be useful human cGAS inhibitors; however, they also require further investigation (Lama et al. 2019).

#### Inhibitors that disrupt DNA binding

##### Antimalarial drugs (AMDs)

Antimalarial drugs, including quinacrine (QC), 9-amino-6-chloro-2-methoxyacridine (ACMA), and hydroxychloroquine (HCQ), can interfere with cGAS- and dsDNA-binding (An et al. 2015). HCQ can inhibit cGAS activity by nonspecifically binding aminoquinoline and aminoacridine, which occupy the Arg342 and Lys372 DNA-binding sites. QC was found to be the most potent inhibitor of cGAMP (IC_50_ = 13 µM) and IFN-β (IC_50_ = 3.7 µM) production among antimalarial drugs (AMDs) (An et al. 2015). Administration of X6, a novel antimalarial-like drug of the aminoacridine class, to Trex1-deficient mice was significantly more effective than HCQ in attenuating interferon-stimulated gene (ISG) expression (An et al. 2018). X6 was superior to HCQ in inhibiting ISG expression in vitro in peripheral blood mononuclear cells (PBMCs) from systemic lupus erythematosus (SLE) patients. Owing to AMD's excellent safety profile and inhibition of cGAS, the interaction between AMD and cGAS provides a novel therapeutic strategy for the treatment of innate immune diseases.

##### Aspirin

Aspirin, a nonsteroidal anti-inflammatory drug (NSAID), is known to acetylate proteins such as cyclooxygenase (Roth and Majerus 1975; Vane and Botting 2003). Dai et al. (2019) found that aspirin directly acetylated cGAS at Lys384, Lys394 or Lys414 and efficiently suppressed cGAS-mediated immune responses (Dai et al. 2019). The authors demonstrated that aspirin can effectively inhibit autologous DNA-induced autoimmunity in Aicardi-Goutières syndrome (AGS) patient cells and an AGS mouse model. These findings reveal that cGAS acetylation mediated by aspirin contributes to the regulation of cGAS activity and provides a potential therapy for treating DNA-mediated autoimmune diseases. Aspirin is a widely used drug with approximately 2,200 clinical trials registered on the NIH list. Aspirin has been used in clinical trials (NCT04132791) to prevent and treat cardiovascular disease. Studies evaluating the effects of aspirin on disease recurrence and survival after the first treatment in common nonmetastatic solid tumors are ongoing (NCT02804815).

##### CU-32 and CU-76

Padilla-Salinas et al. (2020) reported a novel drug-binding site for cGAS based on crystallographic studies that revealed the involvement of key residues Lys335 (Lys347 in humans) and Lys382 (Lys394 in humans) in mediating both the cGAS-cGAS protein‒protein interface (PPI) and cGAS-DNA interactions (Padilla-Salinas et al. 2020). Structural docking indicated that the CU family compounds (CU-32 and CU-76), which target the PPI of cGAS, can insert into its zinc capsular structure and inhibit dimer formation through an allosteric effect. Interestingly, CU-32 and CU-76 specifically inhibit the cGAS-STING pathway but do not significantly affect the RIG-I-MAVs pathway or the TLR pathway (Padilla-Salinas et al. 2020). These findings provide a new chemical scaffold and promote the development of new small-molecule inhibitors targeting the human cGAS PPI.

##### ODN A151

A151 is an inhibitory oligodeoxynucleotide containing four repeats of the TTAGGG motif (5′-tt agg gtt agg gtt agg gtt agg g-3′). Steinhagen et al. (2018) reported that A151 can inhibit cGAS activity by interacting with the dsDNA-binding domain in THP-1 human monocytes (Steinhagen et al. 2018). This suppressive activity of A151 depends on both the telomere sequence and the phosphorothioate backbone but represents the first cGAS inhibitor capable of blocking self-DNA. Collectively, these findings may lead to the development of new treatments for IFN-induced pathologies caused by cGAS activation (Steinhagen et al. 2018).

##### Suramin

Suramin, a potent inhibitor of cGAS, was identified by HPLC-based medium-throughput screening (Wang et al. 2018b). Suramin may interfere with the formation of cGAS-dsDNA complexes by displacing bound DNA from cGAS. The inhibition of cGAS by suramin in THP1 cells was selective and did not affect the TLR1/TLR2 or TLR4 pathways (Wang et al. 2018b). The displacement of the DNA in cGAS by suramin or its analogs promotes their use as anti-inflammatory drugs. Currently, there are 21 suramin-related clinical trials on the NIH list, including the suramin study (NCT04496596) in patients with furosemide-resistant AKI.

#### Others

##### Brazilin, and obtusilactone B

Barrier-to-autointegration factor 1 (BAF) was identified as a protein that intrinsically competes with the cGAS component of this pathway for binding to genomic self-DNA (Guey et al. 2020). When nuclear compartmentalization is impaired, cytosolic cGAS enzymatic activity is prevented by BAF. Obtusilactone B, a butanol lactone derivative purified from *spiraea prunifolia*, could inhibit BAF activity (Kim et al. 2013b). The specific binding of obtusilactone B to BAF inhibits vaccinia-associated kinase 1 (VRK1)-mediated BAF phosphorylation, causing DNA nuclear membrane degradation and inactivation of BAF. In addition, Kim et al. isolated brazilin from legumes, which can inhibit BAF phosphorylation in vitro and in vivo by inhibiting VRK1 and disrupting BAF binding to DNA (Kim et al. 2015). Therefore, obtusilactone B and brazilin may be candidates for the indirect regulation of cGAS-STING signaling.

##### Epigallocatechin-3-gallate (EGCG)

GTPase-activating protein SH3 domain-binding protein 1 (G3BP1) promotes the formation of the cGAS complex and enhances cGAS binding to DNA (Liu et al. 2019). Green tea extract epigallocatechin-3-gallate (EGCG), a component extracted from green tea and a G3BP1 inhibitor, has been shown to disrupt the preexisting G3BP1-cGAS complex and inhibit DNA-induced cGAS activation, thus blocking DNA-induced IFN production in vivo and in vitro (Liu et al. 2019). Additionally, EGCG administration impairs the autologous DNA-induced autoinflammatory response in a mouse model of Aicardi-Goutières syndrome (AGS) and reduces IFN-stimulated gene expression in cells from AGS patients. Therefore, EGCG-mediated inhibition of G3BP1 offers a potential treatment for cGAS-associated autoimmune diseases (Liu et al. 2019).

##### Perillaldehyde (PAH)

Perillaldehyde (PAH), a natural monoterpenoid compound derived from Perilla frutescens, suppresses cytoplasmic DNA-induced innate immune responses by inhibiting cGAS activity (Chu et al. 2021). Mice treated with PAH are more susceptible to herpes simplex virus type 1 (HSV-1) infection, and autologous DNA-induced autoinflammatory responses are significantly ameliorated in the AGS mouse model. Although the exact mechanism for PAH inhibition of cGAS remains elusive, PAHs have been demonstrated to effectively inhibit cGAS-STING signaling and can be developed as therapeutics for the treatment of cGAS-mediated autoimmune diseases (Chu et al. 2021).

### STING inhibitors

STING inhibitors can be broadly classified as direct or indirect inhibitors. Direct inhibitors of STING include those targeting the transmembrane domain (TMD) and the ligand-binding domain (LBD) (Fig. 2, Table 2).

#### Direct STING inhibitors

##### Inhibitors targeting the ligand-binding domain

Astin C, a natural cyclic peptide from *Aster tataricus*, inhibits the innate immune CDN sensor STING (Li et al. 2018). Astin C occupies the cGAMP binding pocket by interacting with Ser162, Tyr163 and Arg238 to inhibit human STING (h-STING) function. Based on its high efficacy and low toxicity, astin C can be used to treat STING dysfunction-mediated diseases (Li et al. 2018).

Small molecules (derivatives containing carboxylic acids) were screened to bind to the open conformation of STING in a ratio of 2:1 (Siu et al. 2019). Compound 18 formed a hydrogen bond with Thr263/Thr267 through carboxyl groups and stabilized the open conformation of STING (Siu et al. 2019).

A high-throughput screening approach based on the interaction of small-molecule compounds with recombinant STING proteins was performed (Gao et al. 2022). Interestingly, the cyclin-dependent protein kinase (CDK) inhibitor palbociclib was found to bind directly to STING and inhibit its activation in both mouse macrophages and THP1 cells (Gao et al. 2022). Mechanistically, palbociclib targets Tyr167 of STING and blocks its dimerization, binding to cyclic dinucleotides, and trafficking. Additionally, palbociclib ameliorates autoimmune disease features induced in dextran sodium sulfate (DSS) or Trex1-KO mice (Gao et al. 2022). Thus, palbociclib is a novel pharmacological inhibitor of STING that abrogates the homodimerization of STING and provides a basis for rapid repurposing of FDA-approved drugs for the treatment of autoinflammatory diseases.

Using an in silico docking approach, SN-011 was identified as a potent STING inhibitor that binds to the cyclic dinucleotide (CDN)-binding pocket of STING with higher affinity than endogenous 2′3′-cGAMP (Hong et al. 2021). SN-011 maintains STING in an inactive form, which inhibits the induction of interferon and inflammatory cytokines by 2′3′-cGAMP, herpes simplex virus type 1 infection, Trex1 deficiency, overexpression of cGAS-STING, and STING-associated vasculopathy with onset in infancy (SAVI) mutation (Hong et al. 2021). In Trex1-KO mice, SN-011 was well tolerated, potently suppressed features of inflammatory and autoimmune diseases, and prevented mortality (Hong et al. 2021). Therefore, SN-011, which binds to the STING CDN-binding pocket, is a promising therapeutic agent against STING-induced diseases.

##### Inhibitors targeting the transmembrane domain

Vinogradova et al. (2020) used chemical proteomics to map ligandable cysteines in various immune-related proteins (Vinogradova et al. 2020). Mass spectrometry analysis demonstrated that the active acrylamides BPK-21 and BPK-25 form adducts with Cys91 of STING as well as cysteines of other immune-related proteins. In addition, cytokine secretion related to STING pathway activation was reduced (Vinogradova et al. 2020).

The nitrofuran derivative C-178 and the indole derivative H-151-Al are irreversible inhibitors of mouse and human STING, respectively (Haag et al. 2018). The major inhibitory mechanism is the formation of covalent bonds between C-178 and Cys91 and Cys88 of the STING TMD, which affects the palmitoylation of STING. Nitro fatty acids (NO_2_-FAs/CXA-10) are reported to have inhibitory effects on mouse and human STING (Hansen et al. 2018). NO_2_-FA forms a covalent bond with Cys88/91 and N-terminal His16, which affects the palmitoylation of STING and inhibits TBK1 phosphorylation in fibroblasts derived from patients with STING-associated vascular disease (SAVI). In addition, the STING inhibitor CXA-10 has completed clinical trials (NCT03422510) for its oral use in the treatment of primary focal segmental glomerulosclerosis (FSGS).

#### Indirect STING inhibitors

Compound C is a small-molecule compound that is widely used as an AMPK inhibitor (Zhou et al. 2001). Additionally, Compound C could be used as an inhibitor of the DNA-dependent cGAS-STING pathway (Lai et al. 2020). In vitro assays and liquid chromatography‒mass spectrometry data demonstrate that Compound C has the ability to reduce cGAMP accumulation, indicating that it may function as a modulator in the cGAS-STING-mediated DNA-sensing pathway (Lai et al. 2020). Furthermore, Compound C can rescue the autoimmune phenotype in a mouse model of Trex1 gene deficiency (Lai et al. 2020).

NRF2 inhibits antiviral cytoplasmic sensing by inhibiting the expression of the adapter protein STING (Olagnier et al. 2018). Thus, treating STING-related inflammatory disorders with the NRF2 inducer 4-OI or sulforaphane sufficiently reduced STING-dependent release of type I IFNs from SAVI-derived fibroblasts (Olagnier et al. 2018).

### TBK1 inhibitors

TBK1 is a noncanonical member of the IKK family and binds directly to the CTT of STING oligomers (Zhang et al. 2019). This TBK1 phosphorylates STING and the transcription factor IRF3 to induce type I interferons and other cytokines (Zhang et al. 2019). Therefore, TBK1 is an important mediator of the STING-mediated inflammatory response (Zhao and Zhao 2019). The STING S365A mutation, which prevents IRF3 binding and type I interferon induction, alleviated embryonic lethality in DNase II-/- mice (Li et al. 2022b). The STING S365A mutant, on the other hand, retains the ability to recruit TBK1 and activate NF-κB, and DNase II-/-STING-S365A mice developed severe polyarthritis, which was alleviated by neutralizing antibodies against TNF-α or the IL-6 receptor (Li et al. 2022b). In contrast, the STING L373A mutation or C-terminal tail truncation completely rescued the phenotypes of DNase II-/- mice by disrupting TBK1 binding and subsequently preventing the activation of both IRF3 and NF-κB (Li et al. 2022b). These results demonstrate that TBK1 recruitment to STING mediates autoinflammatory arthritis independent of type I interferons. Candidate drug groups include the TBK1 inhibitors amlexanox (Beyett et al. 2018), compound II (Hasan et al. 2015), curcumin and its analogs (Ullah et al. 2020), GSK8612 (Thomson et al. 2019), luteolin (Lee et al. 2009) and resveratrol (Youn et al. 2005) (Fig. 2, Table 2).

The tyrosine kinase receptor HER2 effectively inhibits cGAS-STING signaling (Wu et al. 2019). Activated HER2 recruits the downstream protein kinase AKT1 and phosphorylates TBK1, blocking the formation of STING and TBK1 complexes and triggering ubiquitination of TBK1, ultimately attenuating STING signaling (Wu et al. 2019). Thus, inhibiting HER2 effectively activates cGAS-STING-mediated signaling. Potential drugs include the small molecule tucatinib, a HER2 inhibitor (Kulukian et al. 2020).

### cGAS activators

cGAS activators have rarely been tested in clinical settings; however, many targeted studies that attempt to overcome tumor immune resistance by activating cGAS are emerging (Table 3).

**Table 3:** Therapeutic application of cGAS and STING activators

Target	Activator		Target diseases	Phase	Clinical trial NCT code
cGAS	Manganese		Unresectable/metastatic solid tumors or lymphomas	Phase I	NCT03991559
	Micronuclei		Senescence and various human cancer cells	Preclinical	
STING	Cyclic Dinucleotides (CDNs)	3′3′-cyclic AIMP	Hepatocellular carcinoma	Preclinical	
		BI 1387446 (BI-STING)	Advanced solid tumors	Phase I	NCT04147234
		BMS-986301	Advanced solid tumors	Phase I	NCT03956680
		E7766	Advanced solid tumors or lymphomas	Phase Ia/Ib	NCT04144140
		exoSTING (CDK-002)	Advanced/metastatic, recurrent, injectable solid tumors	Phase I/ II	NCT04592484
		GSK532	Colorectal carcinoma	Preclinical	
		IMSA101 (GB492)	Refractory malignancies	Phase I/ II	NCT04020185
		JNJ-67544412 (JNJ-4412)	Subcutaneous syngeneic murine tumor models	Preclinical	
		MIW815 (ADU-S100, ML RR-S2 CDA)	Advanced/metastatic solid tumors or lymphomas	Phase I	NCT03172936
			Advanced/metastatic solid tumors or lymphomas	Phase I	NCT02675439
			PD-L1 positive recurrent or metastatic HNSCC	phases II	NCT03937141
		MK-1454	Advanced/metastatic solid tumors or lymphomas	Phase I	NCT03010176
			Metastatic or unresectable, recurrent HNSCC	phase II	NCT04220866
		Nano-STING agonist-decorated microrobot	Murine tumor models	Preclinical	
		SB11285	Advanced solid tumors	Phase Ia/Ib	NCT04096638
	Non-Cyclic Dinucleotides	ALG-031048	Colorectal carcinoma	Preclinical	
		CRD5500 (LB-061)	Colorectal carcinoma	Preclinical	
		CS-1018, CS-1020 and CS-1010	Colon adenocarcinoma and melanoma	Preclinical	
		GSK3745417	Advanced solid tumors	Phase I	NCT03843359
		JNJ- ‘6196	Murine tumor models (not specified)	Preclinical	
		MK-2118	Advanced/metastatic solid tumors or lymphomas	Phase I	NCT03249792
		MSA-1	Colorectal carcinoma	Preclinical	
		MSA-2	Colon adenocarcinoma and melanoma	Preclinical	
		NEs@STING-Mal-NP	TNBC tumor	Preclinical	
		Ryvu’s activators	Colorectal carcinoma	Preclinical	
		SAL-LNP	SARS-CoV-2 infection	Preclinical	
		Selvita activators	In vitro assays for STING activators	Preclinical	
		SNX281	Advanced solid tumors or lymphomas	Phase I	NCT04609579
		TAK-676	Advanced solid tumors	Phase I	NCT04420884
		TTI-10001	Multiple syngeneic murine tumor models	Preclinical	
	Ectonucleotide pyrophosphatase/phosphodiesterase 1(ENPP1) Inhibitor	MV-626	pancreatic adenocarcinoma tumors	Preclinical	
		SR-8314/ SR-8291	Syngeneic murine tumor models	Preclinical	
		SR-8541A	In vitro assays for STING activators	Preclinical	
Others	Bacterial-based activators	STACT	Colorectal carcinoma and melamoma	Preclinical	
		SYNB1891	Advanced solid tumors or lymphomas	Phase I	NCT04167137
	Immune stimulating antibody conjugate (ISAC)	ADC XMT-2056	Advanced/recurrent solid tumors	Phase I	NCT05514717
		TAK-500	Advanced or metastatic solid tumors	Phase Ia/Ib	NCT05070247

#### Manganese (Mn^2+^)

Recent studies have demonstrated that manganese increases the sensitivity of the cGAS-STING pathway to dsDNA and is important for host defenses against DNA viruses and antitumor immune responses (Wang et al. 2018a). Indeed, the first human open-label dose-escalation phase I clinical trial has been conducted (NCT03991559) to evaluate the safety and preliminary efficacy of Mn^2+^ priming anti-PD-1 antibody therapy and chemotherapy (Lv et al. 2020). A completed phase I clinical trial in patients with advanced metastatic solid tumors exhibited promising efficacy, the induction of type I interferon, manageable safety, and recovery response to immunotherapy.

Based on this, a protein-based cGAS-STING nanoagonist (bovine serum albumin (BSA)/ferritin-based nanoagonist incorporating manganese (II) ions and β-lapachone) was discovered to enhance tumor-specific T-cell-mediated immune responses against poorly immunogenic solid tumors in vivo (Wang et al. 2022).

MnO_2_ nanoparticles are a novel cGAS activator that acts as a multifunctional biomaterial with physical, chemical, and biological properties. It can undergo conversion to Mn2^+^ through reactions with intracellular H_2_O_2_ or GSH, making it potentially valuable for the development of new therapies and vaccines for certain diseases, such as tumors and infections (Zhang et al. 2023a).

#### Micronuclei

Micronuclei produced by chromosomal instability in cancer cells activate the cGAS/STING innate immune pathway (Mackenzie et al. 2017). Here, cGAS detects dsDNA inside ruptured micronuclei with fragile envelopes. However, the results of cGAS/STING pathway activation are controversial. Recent reports indicate that cGAS/STING pathway activation promotes tumor metastasis through the activation of the noncanonical NF-kb pathway (Dou et al. 2017; Mackenzie et al. 2017; Bakhoum et al. 2018). However, some reports suggest that cancer cells with elevated cGAS/STING/IRF3 protein levels in tumor progression and metastasis exhibit enhanced cGAS-STING pathway activation, which induces mitochondrial outer membrane permeability and triggers apoptosis (Mitchison et al. 2017; Zierhut et al. 2019).

### STING activators

Since the discovery of STING, numerous natural and synthetic STING activators have been tested in preclinical and clinical settings in various oncological settings (Table 3).

A first-generation STING activator, DMXAA (also named ASA404 and Vadimezan), exhibited efficacy in mouse solid tumors but failed in clinical trials because it did not bind to human STING (Kim et al. 2013a). Since then, various human STING activators have been rapidly developed. STING activators are being developed in the form of cyclic dinucleotides (CDNs), noncyclic dinucleotides, bacterial vectors, immune-stimulating antibody conjugate (ISAC), and macrocyclic STING activators, among others. Recently, nanovaccines (ONM-500 (Miller et al. 2020), neoantigen nanovaccines (Luo et al. 2017; Zhou et al. 2020)) and nanoparticles (STING-NPs) (Wehbe et al. 2021) have been developed and are expected to be rapid developments of new innate immune activations.

#### Cyclic dinucleotides (CDNs)

##### 3′3′-Cyclic AIMP

A CDN STING activator, 3′3′-cyclic AIMP, was developed as a derivative of 2′3′ cyclic GMP-AMP. In a murine model of hepatocellular carcinoma (HCC), when 3′3′-cyclic AIMP was administered at a later stage following HCC development, the tumor size was reduced significantly (Thomsen et al. 2020).

##### BI 1387446 (BI-STING)

BI 1387446 is a BI-STING compound that mimics the natural STING ligand. Preclinical data using SB11285 in oncology mouse models demonstrate significantly higher inhibition of tumor growth in mice injected with intratumoral SB11285 compared with the control group. Additionally, SB11285 in combination with cyclophosphamide resulted in a significant synergistic antitumor effect. A phase I clinical study is currently ongoing and is analyzing BI 1387446 both alone and in combination with ezabenlimab (BI 754091, an anti-PD-1 monoclonal antibody) in patients with different types of advanced and metastatic cancers (NCT04147234) (Gremel et al. 2020).

##### BMS-986301

BMS-986301 is a synthetic STING activator originally developed by IFM Therapeutics and later acquired by Bristol-Myers Squibb. In CT26 or MC38 mouse models, BMS-986301 monotherapy yielded complete regression in 90% of cases. In the CT26 model, the combination of BMS-986301 and anti-PD-1 monoclonal antibody resulted in complete regression in 80% of cases, whereas complete regression was not observed following treatment with anti-PD-1 alone (Schieven et al. 2018). Furthermore, all CT26 mice that exhibited complete tumor regression developed an immunological memory that rejected new tumor cells without further treatment. BMS-986301 is currently undergoing a phase I clinical study (NCT03956680) for intratumoral or intramuscular injection as both a monotherapy and a combination therapy with nivolumab and ipilimumab in patients with advanced solid cancers who have previously failed to respond to checkpoint inhibiting antibodies.

##### E7766

E7766 is a macrocycle-bridged STING activator (MBSA) derivative of MIW815. E7766 exhibits broad pangenotypic activity in all the major human STING variants in human primary cells (Kim et al. 2021). E7766 demonstrated long-lasting antitumor activity without serious side effects in a liver metastatic tumor model. The clinical efficacy of intratumoral injection of E7766 is being evaluated in a phase Ia and Ib clinical trial as a monotherapy in patients with advanced solid tumors or lymphoma (NCT04144140).

##### exoSTING (CDK-002)

Recently, exoSTING, an engineered extracellular vesicle (EV) loaded with CDNs, was designed to compensate for the weaknesses of CDNs. exoSTING enhances the efficacy of CDNs and preferentially activates antigen-presenting cells in the TME. After intratumoral injection, exoSTING remained intratumoral and enhanced local Th1 responses and CD8 + T-cell recruitment, as well as systemic antitumor immunity to tumors (Jang et al. 2021). exoSTING is currently being investigated in a phase 1/2 clinical trial as a single agent for the treatment of multiple solid tumors (head and neck squamous cell carcinoma (HNSCC), triple negative breast cancer (TNBC), anaplastic thyroid carcinoma (ATC) and cutaneous squamous cell carcinoma (cSCC)). Initial data on exoSTING demonstrated evidence of tolerability, immune activation and tumor shrinkage in patients with advanced/metastatic, recurrent, and injectable solid tumors (NCT04592484).

##### GSK532

One of the CDNs, GSK532, activated STING orthologs in cynomolgus monkeys, minipigs, dogs, rats, and mice while also yielding improved stability in human whole blood. In addition, intratumoral injection of GSK532 into mice of the CT26 murine syngeneic model was shown to induce antitumor effects in both injected and uninjected tumors (Yang et al. 2018).

##### IMSA101 (GB492)

IMSA101, a new small molecule cGAMP analog of cGAMP, was developed by ImmuneSensor Therapeutics. Phase I and phase IIa clinical studies win which patients receive either intratumoral IMSA101 alone or in combination with an immune checkpoint inhibitor (ICI) are ongoing (NCT04020185).

##### JNJ-67544412 (JNJ-4412)

JNJ-67544412 (JNJ-4412) is a recently developed CDN STING activator that is reported to bind to all major alleles of human STING with a stronger affinity than other CDNs. In syngeneic mouse tumor models, intratumoral injection of JNJ-4412 results in significant antitumor efficacy, including increased levels of various proinflammatory cytokines in the tumor and the plasma, increased number of CD8 + T cells in the tumors, loss of angiogenesis, and increased apoptosis (Smith et al. 2020).

##### MIW815 (ADU-S100, ML RR-S2 CDA)

MIW815 (ADU-S100, ML RR-S2 CDA) is a synthetic cyclic dinucleotide (CDN) derivative that activates all human STING alleles and murine STING (Corrales et al. 2015). MIW815 is the first CDN drug to undergo clinical trials as a cancer immunotherapy treatment. Preclinical studies of this drug in various mouse tumor models demonstrated tumor-specific T-cell responses and superior antitumor efficacy in both monotherapy and combination therapy. Moreover, its antitumor effect was highly durable. Three clinical trials (NCT03172936, NCT02675439, and NCT03937141) corresponding to phases I and II demonstrated rather unsatisfactory results: no substantial antitumor activity was observed in two clinical studies (NCT02675439 and NCT03937141) using either monotherapy or combination therapy with ipilimumab or the anti-PD-1 spartalizumab (Corrales et al. 2015; Francica et al. 2018; Sivick et al. 2018). In addition, 12.2% of the reported treatment-related adverse events (TRAEs), including increased lipase levels, diarrhea, and liver dysfunction, were serious and medically significant (grade 3 or 4) (Meric-Bernstam et al. 2022). The pharmaceutical company Novartis withdrew all their clinical trials of MIW815 in December 2019, citing the drug's unsatisfactory clinical efficacy.

##### MK-1454

MK-1454 is the analog of a synthetic CDN developed by Merck & Co. Phase I clinical trials of MK-1454 (NCT03010176) were conducted in participants with advanced/metastatic solid tumors or lymphomas using either MK-1454 alone or MK-1454 in combination with pembrolizumab. The preliminary results illustrated no complete or partial responses in the monotherapy group. However, the combination therapy group exhibited a partial response in 24% (6 of 25) of the patients, which continued for more than six months. Additionally, the median lesion size of the injection site and noninjection site was observed to decrease by 83% (Harrington et al. 2018). To investigate the clinical efficacy of intratumoral MK-1454, a phase II clinical trial was completed in patients with metastatic or unresectable recurrent head and neck squamous cell carcinoma (HNSCC) using monotherapy or combination therapy with pembrolizumab (NCT04220866). The results of the clinical trials are currently being evaluated (McIntosh et al. 2022).

##### Nano-STING agonist-decorated microrobot

Microrobots are composed of VNP bacteria loaded with nano STING activators. The ferric ion-gallic acid (GA) complex, along with cGAMP, is loaded into the inner hydrophilic core, forming cGAMP-Fe@mTNP (mTNP = mitochondria-targeting nanoparticle) nanoagonists via the double-emulsion method. This targets mitochondria and induces mitochondrial oxidative damage through the Fenton reaction, leading to the release of mtDNA. Released mtDNA, cGAMP, and bacteria activate the cGAS/STING pathway in immune cells, triggering the activation of both innate and adaptive immunity, resulting in enhanced anti-tumor efficacy (Wang et al. 2023b).

##### SB11285

SB11285 is a small-molecule CDN STING activator developed by Spring Bank Pharmaceuticals as an anticancer treatment. Compared with cGAMP, SB11285 induced IFN-β with a 200-fold increased potency. Moreover, the drug exhibited very potent and highly durable antitumor activities in vivo when administered intratumorally or intraperitoneally in A20 and CT26 rat cancer models (Challa et al. 2017). Phase Ia and Ib clinical trials are being performed in patients with advanced solid tumors (NCT04096638) to identify the safety, tolerability, and early antitumor activities of intravenously administered SB11285, either alone or in combination with atezolizumab.

#### Noncyclic dinucleotides

##### ALG-031048

ALG-031048 is a novel STING activator that exhibited higher stability in in vitro studies than did the natural STING ligand and STING activator ADU-S100. Intratumoral injection of ALG-031048 into mice bearing CT26 tumor cells resulted in tumor regression in 90% of the mice (compared to 44% for ADU-S100). Moreover, the treated mice were found to be resistant to tumor development following a rechallenge with the same tumor cell line (Jekle et al. 2020b). A further study demonstrated that intratumoral injection of ALG-031048 into mice with Hepa1-6 hepatocellular carcinoma tumor cells resulted in a mean tumor regression of 88% compared with 72.4% regression after treatment with an anti-PD1 antibody. Furthermore, ALG-031048 treatment induced a dose-dependent increase in cytokine levels. Additionally, treatments using a combination of ALG-031048 with the anti-PDL-1 agent atezolizumab further enhanced tumor growth inhibition from 60% with atezolizumab alone to 77% (Jekle et al. 2020a).

##### CRD5500 (LB-061)

The next-generation small molecule STING agonist CRD-5500 was shown to be effective via intratumoral and systemic routes, in addition to an antibody‒drug conjugate (ADC) with trastuzumab. In preclinical studies, both intravenous and intratumoral injections of CRD-5500 induced tumor regression in murine CT26 colon carcinoma models. Its antitumor effect was amplified when CRD-5500 was combined with checkpoint inhibitor therapy (Banerjee et al. 2019).

##### CS-1018, CS-1020, and CS-1010

The CS-1018, CS-1020, and CS-1010 compounds are reported to activate mouse and human STING variants in vitro with a higher potency than the natural ligand cGAMP and reference compounds. All of these compounds demonstrated dose-dependent robust antitumor activity in MC38 and B16F10 syngeneic models. In addition, following treatment with these compounds, tumor-free mice in the MC38 murine model exhibit a tumor-specific immunologic memory response (Li et al. 2020a).

##### GSK3745417

GSK3745417 was developed by GlaxoSmithKline and is a non-CDN small molecule with a dimeric amidobenzimidazole (ABZI) scaffold. Adam et al. measured a panel of 93 tumor cell lines treated with GSK3745417 and reported cytokine production across all cell lines; however, cell growth inhibition occurred only in three cell lines (Adam et al. 2022). Phase I clinical trials have been ongoing since 2019 to analyze the safety, tolerability, and preliminary efficacy of GSK-3745417 and to establish the optimal intravenous therapy in 300 participants with refractory and relapsed solid tumors (NCT03843359). This trial comprised two treatment arms, one in which intravenous GSK-374517 is administered as a monotherapy or and one with GSK-374517 administered in combination with pembrolizumab. Another phase I clinical trial is also underway to test the safety, tolerability, pharmacokinetics, and clinical efficacy of this medicine. Thus, GSK-374517 is being intravenously administered to participants with relapsed or refractory myeloid malignancies, including acute myeloid leukemia (AML) and high-risk myelodysplastic syndrome (HR-MDS).

##### JNJ- ‘6196

JNJ-'6196 was developed as a STING activator that can activate dendritic cells with higher efficacy than other CDNs and induce strong cytokine expression, although its affinity for STING protein is weak and its off rate is fast. In a study using a mouse tumor model, intravenous administration of JNJ-'6196 effectively removed bilateral tumors and promoted immune-mediated resistance to any tumor rechallenge. JNJ-'6196 was also shown to improve the efficacy of checkpoint inhibitors in PD-1 nonresponsive tumor models. The efficacy of JNJ-'6196 in synergistically improving the effects of ICIs increases the feasibility of this compound as a candidate for further clinical development (Chan et al. 2020).

##### MK-2118

MK-2118 was developed by Merck & Co. Currently, MK-2118 is in phase I clinical trials in participants with advanced solid tumors or lymphomas (NCT03249792) to assess its safety and tolerability. Moreover, the maximum tolerated dose (MTD) of MK-2118 will be determined after it has been administered as an intratumoral or subcutaneous injection, either alone or in combination with pembrolizumab (MK-3475) (Sharp and Dohme 2017).

##### MSA-1

The STING activator compound MSA-1 has exhibited robust antitumor efficacy when injected intratumorally into mice possessing MC38 syngeneic colon carcinomas. Complete responses were observed in 100% of the tumors in mice receiving the highest tolerated dose of intratumoral MSA-1. A combination of MSA-1 with an anti-PD1 antibody (mDX400) further promoted the restoration of T-cell responses in anti-PD1-unresponsive tumors. Moreover, this treatment was found to promote the synergistic antitumor activity of STING activators with anti-PD1 therapy (Perera et al. 2018).

##### MSA-2

MSA-2 was discovered following a phenotypic screening process for chemical inducers of interferon-β secretion. Cell-free assays demonstrated that MSA-2 bound to human and mouse STING. A small molecule STING activator that is available orally, MSA-2, has been demonstrated to be an effective activator that can induce tumor regression in mice and produce sustained antitumor immunity alongside activation of IFN-β secretion in a variety of syngeneic murine tumor models. The treatments that use a combination of MSA-2 and anti-PD1 antibodies were found to be advantageous in inhibiting tumor growth and improved the overall survival rate compared with standard component monotherapies (Pan et al. 2020).

##### NEs@STING-Mal-NP

The neotype neutrophil cytopharmaceutical (NEs@STING-Mal-NP), with liposomal STING agonists conjugated on the surface of neutrophils, significantly enhanced the tumor penetration of STING agonists. Additionally, the backpacked liposomal STING agonists were efficiently absorbed by tumor-infiltrating immune cells and tumor cells in response to the abundant hyaluronidase in the tumor environment. Therefore, NEs@STING-Mal-NP effectively activated the STING pathway, converted macrophages and neutrophils into antitumor phenotypes, promoted dendritic cell maturation, and enhanced T cell infiltration and tumoricidal ability. Specifically, when combined with ICI, this cytopharmaceutical exhibited significant inhibition of tumor growth and prolonged the survival of mice with TNBC tumors (Hao et al. 2023).

##### Ryvu’s activators

Using FTS, MST, FP, and crystallographic studies, activators of Ryvu reportedly bind to recombinant STING proteins and selectively activate STING-dependent signaling in both mouse and human immune cells, promoting antitumor immunity. Systemic administration of these compounds in CT26 mouse models bearing colorectal cancer cells resulted in complete tumor regression and the development of immunologic memory (Chmielewski et al. 2020).

##### SAL-LNPs

SAL-LNPs are Synthesized non-nucleotide STING agonist-derived amino lipid-lipid nanoparticles. SAL12-LNPs-mRNA vaccine was identified as the most potent in delivering mRNA encoding the spike glycoprotein (S) of SARS-CoV-2 while activating the STING pathway in dendritic cells (DCs). Intramuscular immunization with SAL12 S-LNPs, administered twice, elicited a robust neutralizing antibody response against SARS-CoV-2 in mice (Zhang et al. 2023b).

##### Selvita activators

The recently developed Selvita activators are a group of small molecule, nonnucleotide, nonmacrocyclic STING activators. These activators selectively bind to both mouse and human STING proteins. The tunable properties of Selvita activators with improved plasma stability and permeability make these activators potential candidates for systemic delivery. In vitro studies on peripheral blood mononuclear cells and the THP1 monocytic cell line indicate that Selvita activators can induce the expression of inflammatory cytokines and upregulate maturation markers on the surface of APCs (Dobrzańska et al. 2019).

##### SNX281

SNX281 is a novel small molecule activator of human and mammalian STING with pharmacokinetic advantages enabling systemic intravenous administration (Wang et al. 2021). This property is due to the mechanism by which the molecule dimerizes at the binding site of STING and induces its activation. In preclinical studies, a single intravenous dose of SNX281 in 26 mice bearing colorectal tumors resulted in complete regression of the tumors. Furthermore, SNX281 synergized with anti-PD-1 agents to inhibit tumor growth and increase the viability of tumor-bearing mice. Indeed, phase I clinical trials are currently ongoing in participants with advanced solid tumors and lymphomas (NCT04609579) to identify and evaluate the safety, tolerability, and maximum tolerated dose of systemic SNX281.

##### TAK-676

TAK-676 is another small molecule STING activator with an unpublished structure that is currently under investigation in a phase I dose-escalation study. This clinical trial aims to determine the safety and tolerability of intravenous TAK-676 as a monotherapy and in combination with pembrolizumab in patients with advanced or metastatic solid cancer (NCT04420884).

##### TTI-10001

TTI-10001 is a non-CDN small molecule STING activator that binds the murine STING protein as well as all five human STING alleles. Intratumoral administration of TTI-10001 was confirmed to be safe in a mouse model of syngeneic tumors. Moreover, TTI-10001 therapy was found to be associated with increased levels of phospho-STING, phospho-IRF3, proinflammatory cytokines, and antitumor activity (Wang et al. 2019b).

#### Ectonucleotide pyrophosphatase/phosphodiesterase 1 (ENPP1) inhibitor

ENPP1 is a transmembrane phosphodiesterase known to play a central role in purinergic signaling (Yegutkin 2008). Recent studies have shown that ENPP1 can downregulate cGAS-STING signaling by hydrolyzing cGAMP, a natural STING ligand (Kato et al. 2018a). Therefore, small molecules that inhibit ENPP1 have been developed as novel STING activators.

##### MV-626

MV-626 is a selective ENPP1 inhibitor with 100% bioavailability and has been studied in preclinical models. In a study in which mice were implanted with Panc02-SIY pancreatic adenocarcinoma tumors, the intraperitoneal injection of MV-626 alone or in combination with radiation therapy resulted in a durable antitumor immune response and improved the overall survival of the mice (Baird et al. 2018).

##### SR-8541A

SR-8541A is a small molecule ENPP1 inhibitor. Recent in vitro studies have demonstrated that SR-8541A can stimulate the migration and infiltration of peripheral blood myeloid cells into the tumor microenvironment (Weston et al. 2020).

##### SR-8314 and SR-8291

SR-8314 and SR-8291, two highly selective ENPP1 inhibitors, have demonstrated robust in vivo efficacy in syngeneic murine tumor models. The intraperitoneal injection of SR-8314 and SR-8291 led to increased frequencies of CD4 + and CD8 + T cells and resulted in a decrease in tumor-associated macrophages in tumor-bearing mice (Weston et al. 2019).

#### Bacterial-based activators

##### STACT

Another bacterial-based immunotherapy, STACT, is an attenuated *Salmonella typhimurium* strain that carries an inhibitory microRNA to TREX-1. The TREX-1 exonuclease prevents the activation of the STING pathway by degrading cytosolic DNA. Preclinical studies illustrated that intravenous injections of STACT-TREX-1 into CT26 and MC38 murine models produced very low systemic levels of inflammatory cytokines and demonstrated tumor-specific colonization, tumor regression, and durable immunity upon rechallenge (Makarova et al. 2019).

##### SYNB1891

SYNB 1891, a nonpathogenic *E. coli* Nissle strain, is a bacterial vector that was engineered to express cyclic di-AMP-producing enzymes in response to the hypoxic environment found in tumors. An intratumoral injection of SYNB1891 into B16. F10 melanoma tumor-bearing mice induced the production of type I IFNs with a concomitant significant reduction in tumor growth eight days after treatment. Phase I clinical trials of SYNB 1981 are currently ongoing in participants with advanced/metastatic solid tumors and lymphoma (NCT04167137) to identify the antitumor efficacy of intratumoral SYNB1891 as both a monotherapy and in combination with atezolizumab (Riese et al. 2021).

#### Immune-stimulating antibody conjugates (ISACs)

##### ADC XMT-2056

ADC XMT-2056 is an antibody‒drug conjugate (ADC) linked to a payload consisting of an anti-human epidermal growth factor receptor 2 (EGFR2; HER2; ErbB2) antibody (HT-19) and an agent for STING, which possess potential immune and antitumor activities. After administering the anti-HER2 STING activator ADC XMT-2056 intravenously, the anti-HER2 antibody moiety targets and binds HER2, while the STING activator targets the immune cells in the tumor microenvironment (TME) and binds to STING. This mechanism leads to the production of proinflammatory cytokines, including interferon (IFN), through the specific activation of the STING pathway in the TME, which promotes the cross-presentation of tumor-associated antigens (TAAs) by dendritic cells (DCs) and induces a cytotoxic T lymphocyte (CTL)-mediated immune response. Conjugation of anti-HER2 antibodies to STING activators improved the targeted delivery of STING activators, increased tumor exposure, and enhanced STING-mediated antitumor immune responses while limiting systemic toxicity (Bukhalid et al. 2020). XMT-2056 entered phase I clinical trials in 2022 (NCT05514717).

##### TAK-500

TAK-500 is an immune stimulatory antibody conjugate (ISAC) that consists of three parts. Cysteine‒cysteine chemokine receptor type 2 (CCR2) is expressed by tumor-infiltrating myeloid cells, including tumor-associated macrophages (TAMs), and promotes immune escape by limiting CD8^+^ T-cell infiltration. TAK-500 demonstrates three therapeutic mechanisms by targeting CCR2-expressing myeloid cells: (1) activation of the IFN response; (2) reprogramming suppressive intratumoral CCR2^+^ cells to an inflammatory phenotype; and (3) blocking suppressive TAM recruitment (Diamond et al. 2022b). A phase Ia and Ib open-label study of TAK-500 (NCT05070247) is currently ongoing in participants aged ≥ 18 years with gastroesophageal adenocarcinoma, pancreatic adenocarcinoma, hepatocellular carcinoma, nonsquamous non-small cell lung cancer, squamous cell carcinoma of the head and neck, mesothelioma, or triple-negative breast cancer (Diamond et al. 2022a).

## Conclusion

In the past few years, knowledge related to the cGAS/STING pathway has expanded widely. Accumulating evidence indicates the critical role of the cGAS/STING pathway in the regulation of immunity leading to the development of immune-related and inflammatory diseases, as cGAS is able to recognize a wide range of both endogenous and exogenous dsDNAs. STING is a convergence point with many upstream receptors in addition to cGAS, suggesting its extensive participation in multiple immune pathways. There has been significant effort to develop both activators and inhibitors for cGAS and STING to enhance or suppress the immune system for appropriate therapeutic purposes. While the activation steps of cGAS/STING during the progression of autoimmune and inflammatory diseases are mostly targeted for the inhibition of these diseases, the lack of cGAS and STING could promote autoinflammation, which requires caution when using cGAS-STING targeted therapy. Recent advances in exploring the mechanisms of the cGAS/STING pathway have played a major role in developing and improving cancer immunotherapy. While chronic activation of cGAS/STING signals can induce an immunosuppressive tumor microenvironment, a few studies have shown that cGAS/STING signals can promote tumor development and metastasis in certain circumstances. Therefore, cGAS-STING targeting therapy needs to be delicately employed depending on the type, characteristics, and metastatic status of cancer.

Understanding the scope and nature of the cGAS/STING pathway and its interactions with other PRRs would highlight its usefulness and lay a roadmap to exploit the pathway as a broad-spectrum therapeutic target.

## Data Availability

Not applicable.

## References

[CR1] Abdullah A, Zhang M, Frugier T, Bedoui S, Taylor JM, Crack PJ (2018). STING-mediated type-I interferons contribute to the neuroinflammatory process and detrimental effects following traumatic brain injury. J Neuroinflammation.

[CR2] Adam M, Yu J, Plant R, Shelton C, Schmidt H, Yang J (2022). Sting agonist GSK3745417 induces apoptosis, antiproliferation, and cell death in a panel of human AML cell lines and patient samples. Blood.

[CR3] Ahn J, Gutman D, Saijo S, Barber GN (2012). STING manifests self DNA-dependent inflammatory disease. Proc Natl Acad Sci U S A.

[CR4] Ahn J, Xia T, Konno H, Konno K, Ruiz P, Barber GN (2014). Inflammation-driven carcinogenesis is mediated through STING. Nat Commun.

[CR5] An J, Woodward JJ, Sasaki T, Minie M, Elkon KB (2015). Cutting edge: antimalarial drugs inhibit IFN-β production through blockade of cyclic GMP-AMP synthase–DNA interaction. J Immunol.

[CR6] An J, Durcan L, Karr RM, Briggs TA, Rice GI, Teal TH, Woodward JJ, Elkon KB (2017). Expression of cyclic GMP-AMP synthase in patients with systemic lupus erythematosus. Arthritis Rheumatol.

[CR7] An J, Woodward JJ, Lai W, Minie M, Sun X, Tanaka L, Snyder JM, Sasaki T, Elkon KB (2018). Inhibition of cyclic GMP-AMP synthase using a novel antimalarial drug derivative in Trex1-deficient mice. Arthritis Rheumatol.

[CR8] An X, Zhu Y, Zheng T, Wang G, Zhang M, Li J, Ji H, Li S, Yang S, Xu D, Li Z, Wang T, He Y, Zhang L, Yang W, Zhao R, Hao D, Li X (2019). An analysis of the expression and association with immune cell infiltration of the cGAS/STING pathway in Pan-cancer. Mol Ther Nucleic Acids.

[CR9] Andreeva L, Hiller B, Kostrewa D, Lässig C, De Oliveira Mann CC, Jan Drexler D, Maiser A, Gaidt M, Leonhardt H, Hornung V, Hopfner KP (2017). cGAS senses long and HMGB/TFAM-bound U-turn DNA by forming protein-DNA ladders. Nature.

[CR10] Andtbacka RH, Kaufman HL, Collichio F, Amatruda T, Senzer N, Chesney J, Delman KA, Spitler LE, Puzanov I, Agarwala SS, Milhem M, Cranmer L, Curti B, Lewis K, Ross M, Guthrie T, Linette GP, Daniels GA, Harrington K, Middleton MR, Miller WH, Zager JS, Ye Y, Yao B, Li A, Doleman S, Vanderwalde A, Gansert J, Coffin RS (2015). Talimogene laherparepvec improves durable response rate in patients with advanced melanoma. J Clin Oncol.

[CR11] Bai J, Cervantes C, Liu J, He S, Zhou H, Zhang B, Cai H, Yin D, Hu D, Li Z, Chen H, Gao X, Wang F, O'connor JC, Xu Y, Liu M, Dong LQ, Liu F (2017). DsbA-L prevents obesity-induced inflammation and insulin resistance by suppressing the mtDNA release-activated cGAS-cGAMP-STING pathway. Proc Natl Acad Sci USA.

[CR12] Baird JR, Dietsch GN, Florio VA, Gallatin MW, Knox CD, Odingo JO, Crittenden MR, Gough MJ (2018) MV-626, a potent and selective inhibitor of ENPP1 enhances STING activation and augments T-cell mediated anti-tumor activity in vivo. Immunotherapy of cancer 2018 annual meeting

[CR13] Bakhoum SF, Ngo B, Laughney AM, Cavallo JA, Murphy CJ, Ly P, Shah P, Sriram RK, Watkins TBK, Taunk NK, Duran M, Pauli C, Shaw C, Chadalavada K, Rajasekhar VK, Genovese G, Venkatesan S, Birkbak NJ, Mcgranahan N, Lundquist M, Laplant Q, Healey JH, Elemento O, Chung CH, Lee NY, Imielenski M, Nanjangud G, Pe'er D, Cleveland DW, Powell SN, Lammerding J, Swanton C, Cantley LC (2018). Chromosomal instability drives metastasis through a cytosolic DNA response. Nature.

[CR14] Banerjee M, Basu S, Middya S, Shrivastava R, Ghosh R, Pryde DC, Yadav D, Bhattacharya G, Soram T, Puniya K, Raina R, Kadam V, Garai S, Sharma P, Singh A, Shinde V, Rawat N, Middya A, Sinha A, Chandel S, Narisipuram G, Chatterjee A, Mane N, Surya A (2019). Abstract LB-061: CRD5500: a versatile small molecule STING agonist amenable to bioconjugation as an ADC. Cancer Res.

[CR15] Benmerzoug S, Rose S, Bounab B, Gosset D, Duneau L, Chenuet P, Mollet L, Le Bert M, Lambers C, Geleff S, Roth M, Fauconnier L, Sedda D, Carvalho C, Perche O, Laurenceau D, Ryffel B, Apetoh L, Kiziltunc A, Uslu H, Albez FS, Akgun M, Togbe D, Quesniaux VFJ (2018). STING-dependent sensing of self-DNA drives silica-induced lung inflammation. Nat Commun.

[CR16] Beyett TS, Gan X, Reilly SM, Chang L, Gomez AV, Saltiel AR, Showalter HD, Tesmer JJG (2018). Carboxylic acid derivatives of amlexanox display enhanced potency toward TBK1 and IKKepsilon and reveal mechanisms for selective inhibition. Mol Pharmacol.

[CR17] Bi X, Du C, Wang X, Wang XY, Han W, Wang Y, Qiao Y, Zhu Y, Ran L, Liu Y, Xiong J, Huang Y, Liu M, Liu C, Zeng C, Wang J, Yang K, Zhao J (2021). Mitochondrial damage-induced innate immune activation in vascular smooth muscle cells promotes chronic kidney disease-associated plaque vulnerability. Adv Sci.

[CR18] Bouis D, Kirstetter P, Arbogast F, Lamon D, Delgado V, Jung S, Ebel C, Jacobs H, Knapp AM, Jeremiah N, Belot A, Martin T, Crow YJ, Andre-Schmutz I, Korganow AS, Rieux-Laucat F, Soulas-Sprauel P (2019). Severe combined immunodeficiency in stimulator of interferon genes (STING) V154M/wild-type mice. J Allergy Clin Immunol.

[CR19] Bukhalid RA, Duvall JR, Cetinbas NM, Catcott KC, Avocetien K, Bentley KW, Bradley S, Carter T, Chin C-N, Clardy S, Collins SD, Eitas T, Jones BD, Kelleher EW, Mosher R, Nazzaro M, Protopopova M, Shaw P, Slocum K, Ter-Ovanesyan E, Qin L, Thomas JD, Xu L, Yang L, Zurita J, Toader D, Damelin M, Lowinger TB (2020). Abstract 6706: Systemic administration of STING agonist antibody-drug conjugates elicit potent anti-tumor immune responses with minimal induction of circulating cytokines. Can Res.

[CR20] Canesso MCC, Lemos L, Neves TC, Marim FM, Castro TBR, Veloso ES, Queiroz CP, Ahn J, Santiago HC, Martins FS, Alves-Silva J, Ferreira E, Cara DC, Vieira AT, Barber GN, Oliveira SC, Faria AMC (2018). The cytosolic sensor STING is required for intestinal homeostasis and control of inflammation. Mucosal Immunol.

[CR21] Cao DJ, Schiattarella GG, Villalobos E, Jiang N, May HI, Li T, Chen ZJ, Gillette TG, Hill JA (2018). Cytosolic DNA sensing promotes macrophage transformation and governs myocardial ischemic injury. Circulation.

[CR22] Cavagnero KJ, Badrani JH, Naji LH, Amadeo MB, Leng AS, Lacasa LD, Strohm AN, Renusch SR, Gasparian SS, Doherty TA (2021). Cyclic-di-GMP induces STING-dependent ILC2 to ILC1 shift during innate type 2 lung inflammation. Front Immunol.

[CR23] Challa SV, Zhou S, Sheri A, Padmanabhan S, Meher G, Gimi R, Schmidt D, Cleary D, Afdhal N, Iyer R (2017). Preclinical studies of SB 11285, a novel STING agonist for immuno-oncology. J Clin Oncol.

[CR24] Chan TK, Loh XY, Peh HY, Tan WNF, Tan WSD, Li N, Tay IJJ, Wong WSF, Engelward BP (2016). House dust mite-induced asthma causes oxidative damage and DNA double-strand breaks in the lungs. J Allergy Clin Immunol.

[CR25] Chan SR, Bignan G, Pierson E, Mahady S, Ta H, Schepens W, Thuring JW, Lim HK, Otieno M, Wilde T, Singer M, Bogdan N, Patel S, Luistro L, Campion L, Smith M, Wiley D, Packman K, Allegrezza M, Morgan C, Sendecki J, Van Aller G, Krosky D, Connolly P, Edwards J, Staquet K, Emanuel SL (2020). Abstract 5567A: JNJ-‘6196: a next generation STING agonist with potent preclinical activity by the IV route. Can Res.

[CR26] Chen Q, Boire A, Jin X, Valiente M, Er EE, Lopez-Soto A, Jacob L, Patwa R, Shah H, Xu K, Cross JR, Massagué J (2016). Carcinoma–astrocyte gap junctions promote brain metastasis by cGAMP transfer. Nature.

[CR27] Chmielewski S, Zawadzka M, Mazurek J, Rogacki MK, Gluza K, Wójcik-Jaszczyńska K, Poczkaj A, Ćwiertnia G, Topolnicki G, Kujawa M, Zimoląg E, Głowniak-Kwitek U, Mroczkowska M, Gibas A, Leś M, Sudoł S, Wronowski M, Michalik K, Banaszak K, Wiklik K, Malusa F, Combik M, Wiatrowska K, Dudek Ł, Alvarez J, Rajda A, Gajdosz F, Gołas A, Wnuk-Lipińska K, Kuś K, Gabor-Worwa E, Fabritius C, Stasi L, Littlewood P, Brzózka K, Dobrzańska M (2020). Abstract 4532A: development of selective small molecule STING agonists suitable for systemic administration. Cancer Res.

[CR28] Chu L, Li C, Li Y, Yu Q, Yu H, Li C, Meng W, Zhu J, Wang Q, Wang C, Cui S (2021). Perillaldehyde inhibition of cGAS reduces dsDNA-induced interferon response. Front Immunol.

[CR29] Chung KW, Dhillon P, Huang S, Sheng X, Shrestha R, Qiu C, Kaufman BA, Park J, Pei L, Baur J, Palmer M, Susztak K (2019). Mitochondrial damage and activation of the STING pathway lead to renal inflammation and fibrosis. Cell Metab.

[CR30] Ciccia A, Mcdonald N, West SC (2008). Structural and functional relationships of the XPF/MUS81 family of proteins. Annu Rev Biochem.

[CR31] Civril F, Deimling T, De Oliveira Mann CC, Ablasser A, Moldt M, Witte G, Hornung V, Hopfner KP (2013). Structural mechanism of cytosolic DNA sensing by cgas. Nature.

[CR32] Corrales L, Glickman LH, Mcwhirter SM, Kanne DB, Sivick KE, Katibah GE, Woo SR, Lemmens E, Banda T, Leong JJ, Metchette K, Dubensky TW, Gajewski TF (2015). Direct activation of STING in the tumor microenvironment leads to potent and systemic tumor regression and immunity. Cell Rep.

[CR33] Crasta K, Ganem NJ, Dagher R, Lantermann AB, Ivanova EV, Pan Y, Nezi L, Protopopov A, Chowdhury D, Pellman D (2012). DNA breaks and chromosome pulverization from errors in mitosis. Nature.

[CR34] Dai J, Huang Y-J, He X, Zhao M, Wang X, Liu Z-S, Xue W, Cai H, Zhan X-Y, Huang S-Y (2019). Acetylation blocks cGAS activity and inhibits self-DNA-induced autoimmunity. Cell.

[CR35] Demaria O, De Gassart A, Coso S, Gestermann N, Di Domizio J, Flatz L, Gaide O, Michielin O, Hwu P, Petrova TV, Martinon F, Modlin RL, Speiser DE, Gilliet M (2015). STING activation of tumor endothelial cells initiates spontaneous and therapeutic antitumor immunity. Proc Natl Acad Sci USA.

[CR36] Deng Z, Chong Z, Law CS, Mukai K, Ho FO, Martinu T, Backes BJ, Eckalbar WL, Taguchi T, Shum AK (2020). A defect in COPI-mediated transport of STING causes immune dysregulation in COPA syndrome. J Exp Med.

[CR37] Diamond JR, Henry JT, Falchook GS, Olszanski AJ, Singh H, Leonard EJ, Gregory RC, Appleman VA, Gibbs J, Harbison C, Li C, Sapiro JM, Yoneyama T, Parent AA, Chung V (2022). Phase 1a/1b study design of the novel STING agonist, immune-stimulating antibody-conjugate (ISAC) TAK-500, with or without pembrolizumab in patients with advanced solid tumors. J Clin Oncol.

[CR38] Diamond JR, Henry JT, Falchook GS, Olszanski AJ, Singh H, Leonard EJ, Gregory RC, Appleman VA, Gibbs JP, Harbison CE, Li C, Sapiro JM, Yoneyama T, Parent A, Chung V (2022). Abstract CT249: First-in-human study of TAK-500, a novel STING agonist immune stimulating antibody conjugate (ISAC), alone and in combination with pembrolizumab in patients with select advanced solid tumors. Cancer Res.

[CR39] Dobrzańska M, Chmielewski S, Zawadzka M, Mazurek J, Gluza K, Wójcik-Jaszczyńska K, Kujawa M, Topolnicki G, Ćwiertnia G, Poczkaj A, Dolata I, Mroczkowska M, Gibas A, Leś M, Sudoł S, Radzimierski A, Michalik K, Sieprawska-Lupa M, Banaszak K, Wiklik K, Malusa F, Combik M, Wiatrowska K, Adamus A, Dudek L, Alvarez J, Fabritius C, Rajda A, Rogacki M, Gajdosz F, Littlewood P, Stasi L, Brzózka K (2019). Abstract 4983: discovery and characterization of next-generation small molecule direct STING agonists. Cancer Res.

[CR40] Dou Z, Ghosh K, Vizioli MG, Zhu J, Sen P, Wangensteen KJ, Simithy J, Lan Y, Lin Y, Zhou Z (2017). Cytoplasmic chromatin triggers inflammation in senescence and cancer. Nature.

[CR41] Ebner S, Sharon N, Ben-Tal N (2003). Evolutionary analysis reveals collective properties and specificity in the C-type lectin and lectin-like domain superfamily. Protein: Struct Funct Bioinf.

[CR42] Elbourne KB, Keisler D, Mcmurray RW (1998). Differential effects of estrogen and prolactin on autoimmune disease in the NZB/NZW F1 mouse model of systemic lupus erythematosus. Lupus.

[CR43] Francica BJ, Ghasemzadeh A, Desbien AL, Theodros D, Sivick KE, Reiner GL, Hix Glickman L, Marciscano AE, Sharabi AB, Leong ML, Mcwhirter SM, Dubensky TW, Pardoll DM, Drake CG (2018). TNFalpha and radioresistant stromal cells are essential for therapeutic efficacy of cyclic dinucleotide STING agonists in nonimmunogenic tumors. Cancer Immunol Res.

[CR44] Fu J, Kanne DB, Leong M, Glickman LH, Mcwhirter SM, Lemmens E, Mechette K, Leong JJ, Lauer P, Liu W (2015). STING agonist formulated cancer vaccines can cure established tumors resistant to PD-1 blockade. Science Translational Medicine.

[CR45] Gao D, Li T, Li XD, Chen X, Li QZ, Wight-Carter M, Chen ZJ (2015). Activation of cyclic GMP-AMP synthase by self-DNA causes autoimmune diseases. Proc Natl Acad Sci USA.

[CR46] Gao J, Zheng M, Wu X, Zhang H, Su H, Dang Y, Ma M, Wang F, Xu J, Chen L, Liu T, Chen J, Zhang F, Yang L, Xu Q, Hu X, Wang H, Fei Y, Chen C, Liu H (2022). CDK inhibitor Palbociclib targets STING to alleviate autoinflammation. EMBO Rep.

[CR47] Gijon Mancheno A, Jansen W, Winterwerp JC, Uijttewaal WSJ (2021). Predictive model of bulk drag coefficient for a nature-based structure exposed to currents. Sci Rep.

[CR48] Gray EE, Treuting PM, Woodward JJ, Stetson DB (2015). Cutting edge: cGAS is required for lethal autoimmune disease in the Trex1-deficient mouse model of Aicardi-Goutieres syndrome. J Immunol.

[CR49] Gremel G, Impagnatiello MA, Carotta S, Schaaf O, Chetta PM, Oost T, Zichner T, Hofmann M, Blake S, Bretschneider T, Fleck M, Grube A, Nar H, Rast G, Schmidt E, Klinkhardt U, Arndt-Schmitz K, Laux T, Zinzalla V, Sedgwick J, Kraut N (2020). Abstract 4522: potent induction of a tumor-specific immune response by a cyclic dinucleotide STING agonist. Cancer Res.

[CR50] Guey B, Wischnewski M, Decout A, Makasheva K, Kaynak M, Sakar MS, Fierz B, Ablasser A (2020). BAF restricts cGAS on nuclear DNA to prevent innate immune activation. Science.

[CR51] Gui X, Yang H, Li T, Tan X, Shi P, Li M, Du F, Chen ZJ (2019). Autophagy induction via STING trafficking is a primordial function of the cGAS pathway. Nature.

[CR52] Gunther C, Berndt N, Wolf C, Lee-Kirsch MA (2015). Familial chilblain lupus due to a novel mutation in the exonuclease III domain of 3′ repair exonuclease 1 (TREX1). JAMA Dermatol.

[CR53] Haag SM, Gulen MF, Reymond L, Gibelin A, Abrami L, Decout A, Heymann M, Van Der Goot FG, Turcatti G, Behrendt R (2018). Targeting STING with covalent small-molecule inhibitors. Nature.

[CR54] Hall J, Ralph EC, Shanker S, Wang H, Byrnes LJ, Horst R, Wong J, Brault A, Dumlao D, Smith JF, Dakin LA, Schmitt DC, Trujillo J, Vincent F, Griffor M, Aulabaugh AE (2017). The catalytic mechanism of cyclic GMP-AMP synthase (cGAS) and implications for innate immunity and inhibition. Protein Sci.

[CR55] Han Y, Chen L, Liu H, Jin Z, Wu Y, Wu Y, Li W, Ying S, Chen Z, Shen H, Yan F (2020). Airway epithelial cGAS is critical for induction of experimental allergic airway inflammation. J Immunol.

[CR56] Hansen AL, Buchan GJ, Ruhl M, Mukai K, Salvatore SR, Ogawa E, Andersen SD, Iversen MB, Thielke AL, Gunderstofte C, Motwani M, Moller CT, Jakobsen AS, Fitzgerald KA, Roos J, Lin R, Maier TJ, Goldbach-Mansky R, Miner CA, Qian W, Miner JJ, Rigby RE, Rehwinkel J, Jakobsen MR, Arai H, Taguchi T, Schopfer FJ, Olagnier D, Holm CK (2018). Nitro-fatty acids are formed in response to virus infection and are potent inhibitors of STING palmitoylation and signaling. Proc Natl Acad Sci USA.

[CR57] Hao M, Zhu L, Hou S, Chen S, Li X, Li K, Zhu N, Chen S, Xue L, Ju C, Zhang C (2023). Sensitizing tumors to immune checkpoint blockage via STING agonists delivered by tumor-penetrating neutrophil cytopharmaceuticals. ACS Nano.

[CR58] Harding SM, Benci JL, Irianto J, Discher DE, Minn AJ, Greenberg RA (2017). Mitotic progression following DNA damage enables pattern recognition within micronuclei. Nature.

[CR59] Harrington KJ, Brody J, Ingham M, Strauss J, Cemerski S, Wang M, Tse A, Khilnani A, Marabelle A, Golan T (2018). Preliminary results of the first-in-human (FIH) study of MK-1454, an agonist of stimulator of interferon genes (STING), as monotherapy or in combination with pembrolizumab (pembro) in patients with advanced solid tumors or lymphomas. Ann Oncol.

[CR60] Hasan M, Dobbs N, Khan S, White MA, Wakeland EK, Li QZ, Yan N (2015). Cutting edge: inhibiting TBK1 by compound II ameliorates autoimmune disease in mice. J Immunol.

[CR61] Ho SS, Zhang WY, Tan NY, Khatoo M, Suter MA, Tripathi S, Cheung FS, Lim WK, Tan PH, Ngeow J, Gasser S (2016). The DNA structure-specific endonuclease MUS81 mediates DNA sensor STING-dependent host rejection of prostate cancer cells. Immunity.

[CR62] Hong Z, Mei J, Li C, Bai G, Maimaiti M, Hu H, Yu W, Sun L, Zhang L, Cheng D, Liao Y, Li S, You Y, Sun H, Huang J, Liu X, Lieberman J, Wang C (2021). STING inhibitors target the cyclic dinucleotide binding pocket. Proc Natl Acad Sci USA.

[CR63] Huang YH, Liu XY, Du XX, Jiang ZF, Su XD (2012). The structural basis for the sensing and binding of cyclic di-GMP by STING. Nat Struct Mol Biol.

[CR64] Huffman BJ, Chen S, Schwarz JL, Plata RE, Chin EN, Lairson LL, Houk KN, Shenvi RA (2020). Electronic complementarity permits hindered butenolide heterodimerization and discovery of novel cGAS/STING pathway antagonists. Nat Chem.

[CR65] Inohara N, Nunez G (2003). NODs: intracellular proteins involved in inflammation and apoptosis. Nat Rev Immunol.

[CR66] Janeway CA, Medzhitov R (2002). Innate immune recognition. Ann Rev Immunol.

[CR67] Jang SC, Economides KD, Moniz RJ, Sia CL, Lewis N, Mccoy C, Zi T, Zhang K, Harrison RA, Lim J, Dey J, Grenley M, Kirwin K, Ross NL, Bourdeau R, Villiger-Oberbek A, Estes S, Xu K, Sanchez-Salazar J, Dooley K, Dahlberg WK, Williams DE, Sathyanarayanan S (2021). ExoSTING, an extracellular vesicle loaded with STING agonists, promotes tumor immune surveillance. Commun Biol.

[CR68] Jekle A, Thatikonda S, Stevens S, Williams C, Kinkade A, Ren S, Jaisinghani R, Zhang Q, Misner D, Stoycheva A, Deval J, Mukherjee S, Gonzalvez F, Chanda S, Smith DB, Symons JA, Blatt LM, Beigelman L (2020). Abstract 4520: preclinical characterization of ALG-031048, a novel STING agonist with potent anti-tumor activity in mice. Cancer Res.

[CR69] Jekle A, Thatikonda S, Jaisinghani R, Ren S, Mukherjee S, Chanda S, Symons JA, Blatt LM, Beigelman LN, Gonzalvez F (2020a) Tumor regression in a mouse model of hepatocellular carcinoma upon treatment with the STING agonist ALG-031048. Proceedings of the American association for the study of liver diseases annual meeting.

[CR70] Jounai N, Kobiyama K, Takeshita F, Ishii KJ (2013). Recognition of damage-associated molecular patterns related to nucleic acids during inflammation and vaccination. Front Cell Infect Microbiol.

[CR71] Kato K, Nishimasu H, Oikawa D, Hirano S, Hirano H, Kasuya G, Ishitani R, Tokunaga F, Nureki O (2018). Structural insights into cGAMP degradation by ecto-nucleotide pyrophosphatase phosphodiesterase 1. Nat Commun.

[CR72] Kato Y, Park J, Takamatsu H, Konaka H, Aoki W, Aburaya S, Ueda M, Nishide M, Koyama S, Hayama Y, Kinehara Y, Hirano T, Shima Y, Narazaki M, Kumanogoh A (2018). Apoptosis-derived membrane vesicles drive the cGAS-STING pathway and enhance type I IFN production in systemic lupus erythematosus. Ann Rheum Dis.

[CR73] Khedr S, Dissanayake LV, Palygin O, Staruschenko A (2020). Potential role of cGAS-STING pathway in the induction of diabetic kidney disease. FASEB J.

[CR74] Kim S, Li L, Maliga Z, Yin Q, Wu H, Mitchison TJ (2013). Anticancer flavonoids are mouse-selective STING agonists. ACS Chem Biol.

[CR75] Kim W, Lyu HN, Kwon HS, Kim YS, Lee KH, Kim DY, Chakraborty G, Choi KY, Yoon HS, Kim KT (2013). Obtusilactone B from Machilus Thunbergii targets barrier-to-autointegration factor to treat cancer. Mol Pharmacol.

[CR76] Kim SH, Lyu HN, Kim YS, Jeon YH, Kim W, Kim S, Lim JK, Lee HW, Baek NI, Choi KY, Lee J, Kim KT (2015). Brazilin Isolated from Caesalpinia sappan suppresses nuclear envelope reassembly by inhibiting barrier-to-autointegration factor phosphorylation. J Pharmacol Exp Ther.

[CR77] Kim DS, Endo A, Fang FG, Huang KC, Bao X, Choi HW, Majumder U, Shen YY, Mathieu S, Zhu X, Sanders K, Noland T, Hao MH, Chen Y, Wang JY, Yasui S, Tendyke K, Wu J, Ingersoll C, Loiacono KA, Hutz JE, Sarwar N (2021). E7766, a macrocycle-bridged stimulator of interferon genes (STING) agonist with potent pan-genotypic activity. ChemMedChem.

[CR78] King KR, Aguirre AD, Ye YX, Sun Y, Roh JD, Ng RP, Kohler RH, Arlauckas SP, Iwamoto Y, Savol A, Sadreyev RI, Kelly M, Fitzgibbons TP, Fitzgerald KA, Mitchison T, Libby P, Nahrendorf M, Weissleder R (2017). IRF3 and type I interferons fuel a fatal response to myocardial infarction. Nat Med.

[CR79] Kitajima S, Ivanova E, Guo S, Yoshida R, Campisi M, Sundararaman SK, Tange S, Mitsuishi Y, Thai TC, Masuda S, Piel BP, Sholl LM, Kirschmeier PT, Paweletz CP, Watanabe H, Yajima M, Barbie DA (2019). Suppression of STING associated with LKB1 Loss in KRAS-driven lung cancer. Cancer Discov.

[CR80] Kong L, Li W, Chang E, Wang W, Shen N, Xu X, Wang X, Zhang Y, Sun W, Hu W, Xu P, Liu X (2022). mtDNA-STING axis mediates microglial polarization via IRF3/NF-kappaB signaling after ischemic stroke. Front Immunol.

[CR81] König N, Fiehn C, Wolf C, Schuster M, Cura Costa E, Tüngler V, Alvarez HA, Chara O, Engel K, Goldbach-Mansky R, Günther C, Lee-Kirsch MA (2017). Familial chilblain lupus due to a gain-of-function mutation in STING. Ann Rheumatic Dis.

[CR82] Kulukian A, Lee P, Taylor J, Rosler R, De Vries P, Watson D, Forero-Torres A, Peterson S (2020). Preclinical activity of HER2-selective tyrosine kinase inhibitor tucatinib as a single agent or in combination with trastuzumab or docetaxel in solid tumor models. Mol Cancer Ther.

[CR83] Kumari M, Wang X, Lantier L, Lyubetskaya A, Eguchi J, Kang S, Tenen D, Roh HC, Kong X, Kazak L, Ahmad R, Rosen ED (2016). IRF3 promotes adipose inflammation and insulin resistance and represses browning. J Clin Invest.

[CR84] Lai J, Luo X, Tian S, Zhang X, Huang S, Wang H, Li Q, Cai S, Chen Q (2020). Compound C reducing interferon expression by inhibiting cGAMP accumulation. Front Pharmacol.

[CR85] Lama L, Adura C, Xie W, Tomita D, Kamei T, Kuryavyi V, Gogakos T, Steinberg JI, Miller M, Ramos-Espiritu L, Asano Y, Hashizume S, Aida J, Imaeda T, Okamoto R, Jennings AJ, Michino M, Kuroita T, Stamford A, Gao P, Meinke P, Glickman JF, Patel DJ, Tuschl T (2019). Development of human cGAS-specific small-molecule inhibitors for repression of dsDNA-triggered interferon expression. Nat Commun.

[CR86] Lara PN, Douillard JY, Nakagawa K, Von Pawel J, Mckeage MJ, Albert I, Losonczy G, Reck M, Heo DS, Fan X, Fandi A, Scagliotti G (2011). Randomized phase III placebo-controlled trial of carboplatin and paclitaxel with or without the vascular disrupting agent vadimezan (ASA404) in advanced non-small-cell lung cancer. J Clin Oncol.

[CR87] Lee JK, Kim SY, Kim YS, Lee WH, Hwang DH, Lee JY (2009). Suppression of the TRIF-dependent signaling pathway of toll-like receptors by luteolin. Biochem Pharmacol.

[CR88] Li T, Chen ZJ (2018). The cGAS–cGAMP–STING pathway connects DNA damage to inflammation, senescence, and cancer. J Exp Med.

[CR89] Li X, Shu C, Yi G, Chaton CT, Shelton CL, Diao J, Zuo X, Kao CC, Herr AB, Li P (2013). Cyclic GMP-AMP synthase is activated by double-stranded DNA-induced oligomerization. Immunity.

[CR90] Li S, Hong Z, Wang Z, Li F, Mei J, Huang L, Lou X, Zhao S, Song L, Chen W, Wang Q, Liu H, Cai Y, Yu H, Xu H, Zeng G, Wang Q, Zhu J, Liu X, Tan N, Wang C (2018). The cyclopeptide astin c specifically inhibits the innate immune CDN sensor STING. Cell Rep.

[CR91] Li A, Song Y, Dong C, Chen X, Yang J (2020). Abstract 3317: discovery of novel STING agonists with robust anti-tumor activity. Cancer Res.

[CR92] Li Q, Cao Y, Dang C, Han B, Han R, Ma H, Hao J, Wang L (2020). Inhibition of double-strand DNA-sensing cGAS ameliorates brain injury after ischemic stroke. EMBO Mol Med.

[CR93] Li C, Shen Q, Zhang P, Wang T, Liu W, Li R, Ma X, Zeng X, Yin Y, Tao K (2021). Targeting MUS81 promotes the anticancer effect of WEE1 inhibitor and immune checkpoint blocking combination therapy via activating cGAS/STING signaling in gastric cancer cells. J Exp Clin Cancer Res.

[CR94] Li R, Lin W, Kuang Y, Wang J, Xu S, Shen C, Qiu Q, Shi M, Xiao Y, Liang L, Xu H (2022). cGAS/STING signaling in the regulation of rheumatoid synovial aggression. Ann Transl Med.

[CR95] Li T, Yum S, Li M, Chen X, Zuo X, Chen ZJ (2022). TBK1 recruitment to STING mediates autoinflammatory arthritis caused by defective DNA clearance. J Exp Med.

[CR96] Liang H, Deng L, Hou Y, Meng X, Huang X, Rao E, Zheng W, Mauceri H, Mack M, Xu M, Fu YX, Weichselbaum RR (2017). Host STING-dependent MDSC mobilization drives extrinsic radiation resistance. Nat Commun.

[CR97] Linggonegoro DW, Song H, Jones KM, Lee PY, Schmidt B, Vleugels RA, Huang JT (2021). Familial chilblain lupus in a child with heterozygous mutation in SAMHD1 and normal interferon signature. Br J Dermatol.

[CR98] Liu Y, Jesus AA, Marrero B, Yang D, Ramsey SE, Sanchez GAM, Tenbrock K, Wittkowski H, Jones OY, Kuehn HS, Lee CR, Dimattia MA, Cowen EW, Gonzalez B, Palmer I, Digiovanna JJ, Biancotto A, Kim H, Tsai WL, Trier AM, Huang Y, Stone DL, Hill S, Kim HJ, St Hilaire C, Gurprasad S, Plass N, Chapelle D, Horkayne-Szakaly I, Foell D, Barysenka A, Candotti F, Holland SM, Hughes JD, Mehmet H, Issekutz AC, Raffeld M, Mcelwee J, Fontana JR, Minniti CP, Moir S, Kastner DL, Gadina M, Steven AC, Wingfield PT, Brooks SR, Rosenzweig SD, Fleisher TA, Deng Z, Boehm M, Paller AS, Goldbach-Mansky R (2014). Activated STING in a vascular and pulmonary syndrome. N Engl J Med.

[CR99] Liu ZS, Cai H, Xue W, Wang M, Xia T, Li WJ, Xing JQ, Zhao M, Huang YJ, Chen S, Wu SM, Wang X, Liu X, Pang X, Zhang ZY, Li T, Dai J, Dong F, Xia Q, Li AL, Zhou T, Liu ZG, Zhang XM (2019). G3BP1 promotes DNA binding and activation of cGAS. Nat Immunol.

[CR100] Liu J, Jia Z, Gong W (2021). Circulating mitochondrial DNA stimulates innate immune signaling pathways to mediate acute kidney injury. Front Immunol.

[CR101] Lu GF, Chen SC, Xia YP, Ye ZM, Cao F, Hu B (2021). Synergistic inflammatory signaling by cGAS may be involved in the development of atherosclerosis. Aging (albany NY).

[CR102] Lu X, Wang X, Cheng H, Wang X, Liu C, Tan X (2023). Anti-triple-negative breast cancer metastasis efficacy and molecular mechanism of the STING agonist for innate immune pathway. Ann Med.

[CR103] Luo M, Wang H, Wang Z, Cai H, Lu Z, Li Y, Du M, Huang G, Wang C, Chen X, Porembka MR, Lea J, Frankel AE, Fu YX, Chen ZJ, Gao J (2017). A STING-activating nanovaccine for cancer immunotherapy. Nat Nanotechnol.

[CR104] Luo X, Li H, Ma L, Zhou J, Guo X, Woo SL, Pei Y, Knight LR, Deveau M, Chen Y, Qian X, Xiao X, Li Q, Chen X, Huo Y, Mcdaniel K, Francis H, Glaser S, Meng F, Alpini G, Wu C (2018). Expression of STING Is increased in liver tissues from patients with NAFLD and promotes macrophage-mediated hepatic inflammation and fibrosis in mice. Gastroenterology.

[CR105] Lv M, Chen M, Zhang R, Zhang W, Wang C, Zhang Y, Wei X, Guan Y, Liu J, Feng K, Jing M, Wang X, Liu YC, Mei Q, Han W, Jiang Z (2020). Manganese is critical for antitumor immune responses via cGAS-STING and improves the efficacy of clinical immunotherapy. Cell Res.

[CR106] Ma H, Kang Z, Foo TK, Shen Z, Xia B (2023). Disrupted BRCA1-PALB2 interaction induces tumor immunosuppression and T-lymphocyte infiltration in HCC through cGAS-STING pathway. Hepatology.

[CR107] Mackenzie KJ, Carroll P, Martin C-A, Murina O, Fluteau A, Simpson DJ, Olova N, Sutcliffe H, Rainger JK, Leitch A (2017). cGAS surveillance of micronuclei links genome instability to innate immunity. Nature.

[CR108] Maekawa H, Inoue T, Ouchi H, Jao TM, Inoue R, Nishi H, Fujii R, Ishidate F, Tanaka T, Tanaka Y, Hirokawa N, Nangaku M, Inagi R (2019). Mitochondrial damage causes inflammation via cGAS-STING signaling in acute kidney injury. Cell Rep.

[CR109] Makarova AM, Iannello A, Rae CS, King B, Besprozvannaya M, Faulhaber J, Skoble J, Thanos CD, Glickman LH (2019). Abstract 5016: STACT-TREX1: a systemically-administered STING pathway agonist targets tumor-resident myeloid cells and induces adaptive anti-tumor immunity in multiple preclinical models. Cancer Res.

[CR110] Mannino DM, Buist AS (2007). Global burden of COPD: risk factors, prevalence, and future trends. Lancet.

[CR111] Mao Y, Luo W, Zhang L, Wu W, Yuan L, Xu H, Song J, Fujiwara K, Abe JI, Lemaire SA, Wang XL, Shen YH (2017). STING-IRF3 triggers endothelial inflammation in response to free fatty acid-induced mitochondrial damage in diet-induced obesity. Arterioscler Thromb Vasc Biol.

[CR112] Mcintosh JA, Liu Z, Andresen BM, Marzijarani NS, Moore JC, Marshall NM, Borra-Garske M, Obligacion JV, Fier PS, Peng F, Forstater JH, Winston MS, An C, Chang W, Lim J, Huffman MA, Miller SP, Tsay FR, Altman MD, Lesburg CA, Steinhuebel D, Trotter BW, Cumming JN, Northrup A, Bu X, Mann BF, Biba M, Hiraga K, Murphy GS, Kolev JN, Makarewicz A, Pan W, Farasat I, Bade RS, Stone K, Duan D, Alvizo O, Adpressa D, Guetschow E, Hoyt E, Regalado EL, Castro S, Rivera N, Smith JP, Wang F, Crespo A, Verma D, Axnanda S, Dance ZEX, Devine PN, Tschaen D, Canada KA, Bulger PG, Sherry BD, Truppo MD, Ruck RT, Campeau LC, Bennett DJ, Humphrey GR, Campos KR, Maddess ML (2022). A kinase-cGAS cascade to synthesize a therapeutic STING activator. Nature.

[CR113] Meric-Bernstam F, Sweis RF, Kasper S, Hamid O, Bhatia S, Dummer R, Stradella A, Long GV, Spreafico A, Shimizu T, Steeghs N, Luke JJ, Mcwhirter SM, Muller T, Nair N, Lewis N, Chen X, Bean A, Kattenhorn L, Pelletier M, Sandhu S (2022). Combination of the STING agonist MIW815 and PD-1 inhibitor spartalizumab in advanced/metastatic solid tumors or lymphomas: an open-label, multicenter, phase Ib study. Clin Cancer Res.

[CR114] Miller J, Luo M, Wang H, Wang Z, Ding X, Campbell A, Almazan J, Chen Z, Gao J, Zhao T (2020). P857 ONM-500—a novel STING-activating therapeutic nanovaccine platform for cancer immunotherapy. J ImmunoTher Cancer.

[CR115] Mitchison TJ, Pineda J, Shi J, Florian S (2017). Is inflammatory micronucleation the key to a successful anti-mitotic cancer drug?. Open Biol.

[CR116] Mitrofanova A, Fontanella A, Tolerico M, Mallela S, Molina David J, Zuo Y, Boulina M, Kim JJ, Santos J, Ge M, Sloan A, Issa W, Gurumani M, Pressly J, Ito M, Kretzler M, Eddy S, Nelson R, Merscher S, Burke G, Fornoni A (2022). Activation of stimulator of IFN genes (STING) causes proteinuria and contributes to glomerular diseases. J Am Soc Nephrol.

[CR117] Mitrofanova A, Fontanella A, Tolerico M, Mallela SK, Molina J, Kim JJ, Burke G, Merscher S, Fornoni A (2022). POS-345 STING activation causes proteinuria in mice and contributes to glomerular disease. Kidney Int Rep.

[CR118] Motwani M, Pawaria S, Bernier J, Moses S, Henry K, Fang T, Burkly L, Marshak-Rothstein A, Fitzgerald KA (2019). Hierarchy of clinical manifestations in SAVI N153S and V154M mouse models. Proc Natl Acad Sci USA.

[CR119] Motwani M, Mcgowan J, Antonovitch J, Gao KM, Jiang Z, Sharma S, Baltus GA, Nickerson KM, Marshak-Rothstein A, Fitzgerald KA (2021). cGAS-STING pathway does not promote autoimmunity in murine models of SLE. Front Immunol.

[CR120] Nascimento M, Gombault A, Lacerda-Queiroz N, Panek C, Savigny F, Sbeity M, Bourinet M, Le Bert M, Riteau N, Ryffel B, Quesniaux VFJ, Couillin I (2019). Self-DNA release and STING-dependent sensing drives inflammation to cigarette smoke in mice. Sci Rep.

[CR121] Olagnier D, Brandtoft AM, Gunderstofte C, Villadsen NL, Krapp C, Thielke AL, Laustsen A, Peri S, Hansen AL, Bonefeld L, Thyrsted J, Bruun V, Iversen MB, Lin L, Artegoitia VM, Su C, Yang L, Lin R, Balachandran S, Luo Y, Nyegaard M, Marrero B, Goldbach-Mansky R, Motwani M, Ryan DG, Fitzgerald KA, O'Neill LA, Hollensen AK, Damgaard CK, De Paoli FV, Bertram HC, Jakobsen MR, Poulsen TB, Holm CK (2018). Nrf2 negatively regulates STING indicating a link between antiviral sensing and metabolic reprogramming. Nat Commun.

[CR122] Padilla-Salinas R, Sun L, Anderson R, Yang X, Zhang S, Chen ZJ, Yin H (2020). Discovery of small-molecule cyclic GMP-AMP synthase inhibitors. J Org Chem.

[CR123] Pan BS, Perera SA, Piesvaux JA, Presland JP, Schroeder GK, Cumming JN, Trotter BW, Altman MD, Buevich AV, Cash B, Cemerski S, Chang W, Chen Y, Dandliker PJ, Feng G, Haidle A, Henderson T, Jewell J, Kariv I, Knemeyer I, Kopinja J, Lacey BM, Laskey J, Lesburg CA, Liang R, Long BJ, Lu M, Ma Y, Minnihan EC, O'Donnell G, Otte R, Price L, Rakhilina L, Sauvagnat B, Sharma S, Tyagarajan S, Woo H, Wyss DF, Xu S, Bennett DJ, Addona GH (2020). An orally available non-nucleotide STING agonist with antitumor activity. Science.

[CR124] Perera SA, Kopinja JE, Ma Y, Laskey J, Chakravarthy K, Chen Y, Cui L, Presland J, Zhao S, Minnihan E, Ferguson H, Piesvaux J, Pan B-S, Woo HC, Knemeyer I, Cemerski S, Cumming J, Trotter W, Tse A, Addona GH, Long BJ (2018). Abstract 4721: combining STING agonists with an anti-PD-1 antagonist results in marked antitumor activity in immune-excluded tumors. Cancer Res.

[CR125] Pouwels SD, Zijlstra GJ, Van Der Toorn M, Hesse L, Gras R, Ten Hacken NH, Krysko DV, Vandenabeele P, De Vries M, Van Oosterhout AJ, Heijink IH, Nawijn MC (2016). Cigarette smoke-induced necroptosis and DAMP release trigger neutrophilic airway inflammation in mice. Am J Physiol Lung Cell Mol Physiol.

[CR126] Qi Z, Yan F, Chen D, Xing W, Li Q, Zeng W, Bi B, Xie J (2020). Biosci Rep.

[CR127] Qiao JT, Cui C, Qing L, Wang LS, He TY, Yan F, Liu FQ, Shen YH, Hou XG, Chen L (2018). Activation of the STING-IRF3 pathway promotes hepatocyte inflammation, apoptosis and induces metabolic disorders in nonalcoholic fatty liver disease. Metabolism.

[CR128] Ranoa DRE, Widau RC, Mallon S, Parekh AD, Nicolae CM, Huang X, Bolt MJ, Arina A, Parry R, Kron SJ, Moldovan G-L, Khodarev NN, Weichselbaum RR (2019). STING promotes homeostasis via regulation of cell proliferation and chromosomal stability. Can Res.

[CR129] Rehwinkel J, Gack MU (2020). RIG-I-like receptors: their regulation and roles in RNA sensing. Nat Rev Immunol.

[CR130] Richeldi L, Collard HR, Jones MG (2017). Idiopathic pulmonary fibrosis. Lancet.

[CR131] Riese R, Luke J, Lewis K, Janku F, Piha-Paul S, Verschraegen C, Brennan A, Armstrong M, Varterasian M, Sokolovska A, Strauss J (2021). 500 SYNB1891, a bacterium engineered to produce a STING agonist, demonstrates target engagement in humans following intratumoral injection. J ImmunoTher Cancer.

[CR132] Roth GJ, Majerus PW (1975). The mechanism of the effect of aspirin on human platelets: I—acetylation of a particulate fraction protein. J Clin Invest.

[CR133] Ryu C, Sun H, Gulati M, Herazo-Maya JD, Chen Y, Osafo-Addo A, Brandsdorfer C, Winkler J, Blaul C, Faunce J, Pan H, Woolard T, Tzouvelekis A, Antin-Ozerkis DE, Puchalski JT, Slade M, Gonzalez AL, Bogenhagen DF, Kirillov V, Feghali-Bostwick C, Gibson K, Lindell K, Herzog RI, Dela Cruz CS, Mehal W, Kaminski N, Herzog EL, Trujillo G (2017). Extracellular mitochondrial DNA is generated by fibroblasts and predicts death in idiopathic pulmonary fibrosis. Am J Respir Crit Care Med.

[CR134] Savigny F, Schricke C, Lacerda-Queiroz N, Meda M, Nascimento M, Huot-Marchand S, Da Gama MF, Ryffel B, Gombault A, Le Bert M, Couillin I, Riteau N (2020). Protective role of the nucleic acid sensor STING in pulmonary fibrosis. Front Immunol.

[CR135] Schieven G, Brown J, Swanson J, Stromko B, Ho C, Zhang R, Li W, Qiu H, Sun H, Fink B (2018) Preclinical characterization of BMS-986301, a differentiated STING agonist with robust antitumor activity as monotherapy or in combination with anti-PD-1. Proceedings of the 33rd annual meeting & pre-conference programs of the society for immunotherapy of cancer (SITC 2018), Washington, DC, pp. 7–11.

[CR136] Schuliga M, Kanwal A, Read J, Blokland KEC, Burgess JK, Prele CM, Mutsaers SE, Grainge C, Thomson C, James A, Bartlett NW, Knight DA (2021). A cGAS-dependent response links DNA damage and senescence in alveolar epithelial cells: a potential drug target in IPF. Am J Physiol Lung Cell Mol Physiol.

[CR137] Seok JK, Kang HC, Cho YY, Lee HS, Lee JY (2021). Therapeutic regulation of the NLRP3 inflammasome in chronic inflammatory diseases. Arch Pharm Res.

[CR138] Shang G, Zhang C, Chen ZJ, Bai XC, Zhang X (2019). Cryo-EM structures of STING reveal its mechanism of activation by cyclic GMP-AMP. Nature.

[CR139] Sharp M, Dohme L (2017) Study of MK-2118 administered as intratumoral injection as monotherapy and in combination with pembrolizumab (MK-3475) or by subcutaneous injection in combination with pembrolizumab in the treatment of adults with advanced/metastatic solid tumors or lymphomas (MK-2118-001). https://ClinicalTrials.gov/show/NCT03249792

[CR140] Siedel H, Roers A, Rosen-Wolff A, Luksch H (2020). Type I interferon-independent T cell impairment in a Tmem173 N153S/WT mouse model of STING associated vasculopathy with onset in infancy (SAVI). Clin Immunol.

[CR141] Siu T, Altman MD, Baltus GA, Childers M, Ellis JM, Gunaydin H, Hatch H, Ho T, Jewell J, Lacey BM, Lesburg CA, Pan BS, Sauvagnat B, Schroeder GK, Xu S (2019). Discovery of a novel cGAMP competitive ligand of the inactive form of STING. ACS Med Chem Lett.

[CR142] Sivick KE, Desbien AL, Glickman LH, Reiner GL, Corrales L, Surh NH, Hudson TE, Vu UT, Francica BJ, Banda T, Katibah GE, Kanne DB, Leong JJ, Metchette K, Bruml JR, Ndubaku CO, Mckenna JM, Feng Y, Zheng L, Bender SL, Cho CY, Leong ML, Van Elsas A, Dubensky TW, Mcwhirter SM (2018). Magnitude of therapeutic STING activation determines CD8(+) T cell-mediated anti-tumor immunity. Cell Rep.

[CR143] Skoulidis F, Goldberg ME, Greenawalt DM, Hellmann MD, Awad MM, Gainor JF, Schrock AB, Hartmaier RJ, Trabucco SE, Gay L, Ali SM, Elvin JA, Singal G, Ross JS, Fabrizio D, Szabo PM, Chang H, Sasson A, Srinivasan S, Kirov S, Szustakowski J, Vitazka P, Edwards R, Bufill JA, Sharma N, Ou SI, Peled N, Spigel DR, Rizvi H, Aguilar EJ, Carter BW, Erasmus J, Halpenny DF, Plodkowski AJ, Long NM, Nishino M, Denning WL, Galan-Cobo A, Hamdi H, Hirz T, Tong P, Wang J, Rodriguez-Canales J, Villalobos PA, Parra ER, Kalhor N, Sholl LM, Sauter JL, Jungbluth AA, Mino-Kenudson M, Azimi R, Elamin YY, Zhang J, Leonardi GC, Jiang F, Wong KK, Lee JJ, Papadimitrakopoulou VA, Wistuba II, Miller VA, Frampton GM, Wolchok JD, Shaw AT, Janne PA, Stephens PJ, Rudin CM, Geese WJ, Albacker LA, Heymach JV (2018). STK11/LKB1 mutations and PD-1 inhibitor resistance in KRAS-mutant lung adenocarcinoma. Cancer Discov.

[CR144] Smith M, Chin D, Chan S, Mahady S, Campion L, Morgan C, Patel S, Chu G, Hughes A, Bignan G, Connolly P, Emanuel S, Packman K, Luistro LL (2020). Abstract 5567: in vivo administration of the STING agonist, JNJ-67544412, leads to complete regression of established murine subcutaneous tumors. Cancer Res.

[CR145] Song S, Peng P, Tang Z, Zhao J, Wu W, Li H, Shao M, Li L, Yang C, Duan F, Zhang M, Zhang J, Wu H, Li C, Wang X, Wang H, Ruan Y, Gu J (2017). Decreased expression of STING predicts poor prognosis in patients with gastric cancer. Sci Rep.

[CR146] Steinhagen F, Zillinger T, Peukert K, Fox M, Thudium M, Barchet W, Putensen C, Klinman D, Latz E, Bode C (2018). Suppressive oligodeoxynucleotides containing TTAGGG motifs inhibit cGAS activation in human monocytes. Eur J Immunol.

[CR147] Sun L, Wu J, Du F, Chen X, Chen ZJ (2013). Cyclic GMP-AMP synthase is a cytosolic DNA sensor that activates the type I interferon pathway. Science.

[CR148] Takeda K, Akira S (2005). Toll-like receptors in innate immunity. Int Immunol.

[CR149] Thim-Uam A, Prabakaran T, Tansakul M, Makjaroen J, Wongkongkathep P, Chantaravisoot N, Saethang T, Leelahavanichkul A, Benjachat T, Paludan S, Pisitkun T, Pisitkun P (2020). STING mediates lupus via the activation of conventional dendritic cell maturation and plasmacytoid dendritic cell differentiation. iScience.

[CR150] Thomsen MK, Skouboe MK, Boularan C, Vernejoul F, Lioux T, Leknes SL, Berthelsen MF, Riedel M, Cai H, Joseph JV, Perouzel E, Tiraby M, Vendelbo MH, Paludan SR (2020). The cGAS-STING pathway is a therapeutic target in a preclinical model of hepatocellular carcinoma. Oncogene.

[CR151] Thomson DW, Poeckel D, Zinn N, Rau C, Strohmer K, Wagner AJ, Graves AP, Perrin J, Bantscheff M, Duempelfeld B, Kasparcova V, Ramanjulu JM, Pesiridis GS, Muelbaier M, Bergamini G (2019). Discovery of GSK8612, a highly selective and potent TBK1 inhibitor. ACS Med Chem Lett.

[CR152] Tse KM, Takeuchi O (2023). Innate immune sensing of pathogens and its post-transcriptional regulations by RNA-binding proteins. Arch Pharm Res.

[CR153] Ullah MA, Johora FT, Sarkar B, Araf Y, Rahman MH (2020). Curcumin analogs as the inhibitors of TLR4 pathway in inflammation and their drug like potentialities: a computer-based study. J Recept Signal Transduct Res.

[CR154] Vane JR, Botting RM (2003). The mechanism of action of aspirin. Thromb Res.

[CR155] Vece TJ, Watkin LB, Nicholas S, Canter D, Braun MC, Guillerman RP, Eldin KW, Bertolet G, Mckinley S, De Guzman M, Forbes L, Chinn I, Orange JS (2016). Copa syndrome: a novel autosomal dominant immune dysregulatory disease. J Clin Immunol.

[CR156] Vincent J, Adura C, Gao P, Luz A, Lama L, Asano Y, Okamoto R, Imaeda T, Aida J, Rothamel K, Gogakos T, Steinberg J, Reasoner S, Aso K, Tuschl T, Patel DJ, Glickman JF, Ascano M (2017). Small molecule inhibition of cGAS reduces interferon expression in primary macrophages from autoimmune mice. Nat Commun.

[CR157] Vinogradova EV, Zhang X, Remillard D, Lazar DC, Suciu RM, Wang Y, Bianco G, Yamashita Y, Crowley VM, Schafroth MA, Yokoyama M, Konrad DB, Lum KM, Simon GM, Kemper EK, Lazear MR, Yin S, Blewett MM, Dix MM, Nguyen N, Shokhirev MN, Chin EN, Lairson LL, Melillo B, Schreiber SL, Forli S, Teijaro JR, Cravatt BF (2020). An activity-guided map of electrophile-cysteine interactions in primary human T Cells. Cell.

[CR158] Wang Z, Celis E (2015). STING activator c-di-GMP enhances the anti-tumor effects of peptide vaccines in melanoma-bearing mice. Cancer Immunol Immunother.

[CR159] Wang L, Xie L, Zhang Q, Cai X, Tang Y, Wang L, Hang T, Liu J, Gong J (2015). Plasma nuclear and mitochondrial DNA levels in acute myocardial infarction patients. Coron Artery Dis.

[CR160] Wang Y, Su GH, Zhang F, Chu JX, Wang YS (2015). Cyclic GMP-AMP synthase is required for cell proliferation and inflammatory responses in rheumatoid arthritis synoviocytes. Mediators Inflamm.

[CR161] Wang C, Guan Y, Lv M, Zhang R, Guo Z, Wei X, Du X, Yang J, Li T, Wan Y, Su X, Huang X, Jiang Z (2018). Manganese Increases the sensitivity of the cGAS-STING pathway for double-stranded DNA and is required for the host defense against DNA viruses. Immunity.

[CR162] Wang M, Sooreshjani MA, Mikek C, Opoku-Temeng C, Sintim HO (2018). Suramin potently inhibits cGAMP synthase, cGAS, in THP1 cells to modulate IFN-beta levels. Future Med Chem.

[CR163] Wang J, Li R, Lin H, Qiu Q, Lao M, Zeng S, Wang C, Xu S, Zou Y, Shi M, Liang L, Xu H, Xiao Y (2019). Accumulation of cytosolic dsDNA contributes to fibroblast-like synoviocytes-mediated rheumatoid arthritis synovial inflammation. Int Immunopharmacol.

[CR164] Wang Z, Dove P, Rosa D, Bossen B, Helke S, Charbonneau M, Brinen L, Dodge K, Lin GH, Galligan C, Viller NN, Wong M, Lee V, Catalano T, Uger RA, Slassi M, Winston J (2019). Abstract 3854: preclinical characterization of a novel non-cyclic dinucleotide small molecule STING agonist with potent antitumor activity in mice. Cancer Res.

[CR165] Wang X, Rao H, Zhao J, Wee A, Li X, Fei R, Huang R, Wu C, Liu F, Wei L (2020). STING expression in monocyte-derived macrophages is associated with the progression of liver inflammation and fibrosis in patients with nonalcoholic fatty liver disease. Lab Invest.

[CR166] Wang J, Falchook G, Nabhan S, Kulkarni M, Sandy P, Dosunmu O, Gardner H, Bendell J, Johnson M (2021). 495 Trial of SNX281, a systemically delivered small molecule STING agonist, in solid tumors and lymphomas. J ImmunoTher Cancer.

[CR167] Wang X, Liu Y, Xue C, Hu Y, Zhao Y, Cai K, Li M, Luo Z (2022). A protein-based cGAS-STING nanoagonist enhances T cell-mediated anti-tumor immune responses. Nat Commun.

[CR168] Wang X, Zhang H, Wang Y, Bramasole L, Guo K, Mourtada F, Meul T, Hu Q, Viteri V, Kammerl I, Konigshoff M, Lehmann M, Magg T, Hauck F, Fernandez IE, Meiners S (2023). DNA sensing via the cGAS/STING pathway activates the immunoproteasome and adaptive T-cell immunity. EMBO J.

[CR169] Wang Y, Li Z, Chen Y, Barrett A, Mo F, Hu Q (2023). Nano-STING agonist-decorated microrobots boost innate and adaptive anti-tumor immunity. Nano Res.

[CR170] Warner JD, Irizarry-Caro RA, Bennion BG, Ai TL, Smith AM, Miner CA, Sakai T, Gonugunta VK, Wu J, Platt DJ, Yan N, Miner JJ (2017). STING-associated vasculopathy develops independently of IRF3 in mice. J Exp Med.

[CR171] Wehbe M, Wang-Bishop L, Becker KW, Shae D, Baljon JJ, He X, Christov P, Boyd KL, Balko JM, Wilson JT (2021). Nanoparticle delivery improves the pharmacokinetic properties of cyclic dinucleotide STING agonists to open a therapeutic window for intravenous administration. J Controlled Release.

[CR172] Weston A, Thode T, Munoz R, Daniel S, Soldi R, Kaadige M, Han H, Vankayalapti H, Sharma S (2019). Abstract 3077: Preclinical studies of SR-8314, a highly selective ENPP1 inhibitor and an activator of STING pathway. Cancer Res.

[CR173] Weston AS, Thode TG, Rodriguez Del Villar R, Dana S, Kasibhatla S, Kaadige MR, Sharma S (2020). Abstract LB-118: SR8541A is a potent inhibitor of ENPP1 and exhibits dendritic cell mediated antitumor activity. Cancer Res.

[CR174] Willemsen J, Neuhoff MT, Hoyler T, Noir E, Tessier C, Sarret S, Thorsen TN, Littlewood-Evans A, Zhang J, Hasan M, Rush JS, Guerini D, Siegel RM (2021). TNF leads to mtDNA release and cGAS/STING-dependent interferon responses that support inflammatory arthritis. Cell Rep.

[CR175] Wilson DR, Sen R, Sunshine JC, Pardoll DM, Green JJ, Kim YJ (2018). Biodegradable STING agonist nanoparticles for enhanced cancer immunotherapy. Nanomed: Nanotechnol Biol Med.

[CR176] Woo S-R, Fuertes MB, Corrales L, Spranger S, Furdyna MJ, Leung MY, Duggan R, Wang Y, Barber GN, Fitzgerald KA (2014). STING-dependent cytosolic DNA sensing mediates innate immune recognition of immunogenic tumors. Immunity.

[CR177] Wu S, Zhang Q, Zhang F, Meng F, Liu S, Zhou R, Wu Q, Li X, Shen L, Huang J, Qin J, Ouyang S, Xia Z, Song H, Feng XH, Zou J, Xu P (2019). HER2 recruits AKT1 to disrupt STING signalling and suppress antiviral defence and antitumour immunity. Nat Cell Biol.

[CR178] Xia T, Konno H, Ahn J, Barber GN (2016). Deregulation of STING signaling in colorectal carcinoma constrains DNA damage responses and correlates with tumorigenesis. Cell Rep.

[CR179] Xia T, Konno H, Barber GN (2016). Recurrent Loss of STING signaling in melanoma correlates with susceptibility to viral OncolysisSTING deregulation in human melanoma. Can Res.

[CR180] Xiong R, Li N, Chen L, Wang W, Wang B, Jiang W, Geng Q (2021). STING protects against cardiac dysfunction and remodelling by blocking autophagy. Cell Commun Signal.

[CR181] Yang J, Adam M, Clemens J, Creech K, Schneck J, Pasikanti K, Tran J-L, Joglekar D, Hopson C, Pesiridis S, Ramanjulu J, Lian Y, Adams JL, Smothers J, Hoos A (2018). Abstract 5554: Preclinical characterization of GSK532, a novel STING agonist with potent anti-tumor activity. Can Res.

[CR182] Yegutkin GG (2008). Nucleotide- and nucleoside-converting ectoenzymes: important modulators of purinergic signalling cascade. Biochim Biophys Acta.

[CR183] Youn HS, Lee JY, Fitzgerald KA, Young HA, Akira S, Hwang DH (2005). Specific inhibition of MyD88-independent signaling pathways of TLR3 and TLR4 by resveratrol: molecular targets are TBK1 and RIP1 in TRIF complex. J Immunol.

[CR184] Yu Y, Liu Y, An W, Song J, Zhang Y, Zhao X (2019). STING-mediated inflammation in Kupffer cells contributes to progression of nonalcoholic steatohepatitis. J Clin Invest.

[CR185] Zang N, Cui C, Guo X, Song J, Hu H, Yang M, Xu M, Wang L, Hou X, He Q, Sun Z, Wang C, Chen L (2022). cGAS-STING activation contributes to podocyte injury in diabetic kidney disease. iScience.

[CR186] Zhang C, Shang G, Gui X, Zhang X, Bai XC, Chen ZJ (2019). Structural basis of STING binding with and phosphorylation by TBK1. Nature.

[CR187] Zhang Y, Chen W, Wang Y (2020). STING is an essential regulator of heart inflammation and fibrosis in mice with pathological cardiac hypertrophy via endoplasmic reticulum (ER) stress. Biomed Pharmacother.

[CR188] Zhang T, Hu C, Zhang W, Ruan Y, Ma Y, Chen D, Huang Y, Fan S, Lin W, Huang Y, Liao K, Lu H, Xu JF, Pi J, Guo X (2023). Advances of MnO(2) nanomaterials as novel agonists for the development of cGAS-STING-mediated therapeutics. Front Immunol.

[CR189] Zhang Y, Yan J, Hou X, Wang C, Kang DD, Xue Y, Du S, Deng B, Mccomb DW, Liu SL, Zhong Y, Dong Y (2023). STING agonist-derived LNP-mRNA vaccine enhances protective immunity against SARS-CoV-2. Nano Lett.

[CR190] Zhao C, Zhao W (2019). TANK-binding kinase 1 as a novel therapeutic target for viral diseases. Expert Opin Ther Targets.

[CR191] Zhao B, Du F, Xu P, Shu C, Sankaran B, Bell SL, Liu M, Lei Y, Gao X, Fu X, Zhu F, Liu Y, Laganowsky A, Zheng X, Ji JY, West AP, Watson RO, Li P (2019). A conserved PLPLRT/SD motif of STING mediates the recruitment and activation of TBK1. Nature.

[CR192] Zhao W, Xiong M, Yuan X, Li M, Sun H, Xu Y (2020). In silico screening-based discovery of novel inhibitors of human cyclic GMP-AMP synthase: a cross-validation study of molecular docking and experimental testing. J Chem Inf Model.

[CR193] Zhou G, Myers R, Li Y, Chen Y, Shen X, Fenyk-Melody J, Wu M, Ventre J, Doebber T, Fujii N, Musi N, Hirshman MF, Goodyear LJ, Moller DE (2001). Role of AMP-activated protein kinase in mechanism of metformin action. J Clin Invest.

[CR194] Zhou W, Whiteley AT, De Oliveira Mann CC, Morehouse BR, Nowak RP, Fischer ES, Gray NS, Mekalanos JJ, Kranzusch PJ (2018). Structure of the human cGAS-DNA complex reveals enhanced control of immune surveillance. Cell.

[CR195] Zhou L, Hou B, Wang D, Sun F, Song R, Shao Q, Wang H, Yu H, Li Y (2020). Engineering polymeric prodrug nanoplatform for vaccination immunotherapy of cancer. Nano Lett.

[CR196] Zierhut C, Yamaguchi N, Paredes M, Luo JD, Carroll T, Funabiki H (2019). The cytoplasmic DNA sensor cGAS promotes mitotic cell death. Cell.

[CR197] Zuk A, Bonventre JV (2016). Acute kidney injury. Annu Rev Med.

